# Sustainable synthesis of quinazolinones: exploring multicomponent reactions with a novel magnetic palladium catalyst

**DOI:** 10.3389/fchem.2026.1632736

**Published:** 2026-04-22

**Authors:** Xiaotong Liang, Ziqi Yang, Bo Li

**Affiliations:** Yangling Vocational & Technical College, Yangling, Shaanxi, China

**Keywords:** Fe 3 O 4 @SiO 2 -Dop/Phen-Pd(0) catalyst, 2-aryl quinazolin-4(3H)-ones, Ecofriendly system, cyclization, carbonylation

## Abstract

This study introduces a sustainable and efficient method for synthesizing quinazolinones, a class of heterocyclic compounds with significant pharmaceutical applications, via a multicomponent reaction (MCR) strategy. The process employs a novel magnetically recoverable palladium catalyst, enabling the coupling of aryl or heteroaryl iodides with a carbonyl source and 2-aminobenzamide in an eco-friendly PEG/water solvent system, facilitated by potassium carbonate as a base. The magnetic Pd catalyst exhibits robust catalytic activity, achieving high product yields (82%–98%) across diverse substrates, including electron-rich and electron-deficient aryl/heteroaryl iodides, underscoring its broad applicability. The catalyst is synthesized and characterized through various techniques, including FT-IR, BET, TGA, EDX, VSM, SEM, TEM, and XRD, which affirm its uniformity and stability. Key advantages of this protocol include exceptional atom economy, elimination of toxic solvents, and mild reaction conditions. The catalyst’s magnetic properties allow effortless recovery via external magnetization, retaining >89% activity over five consecutive cycles, enhancing cost-effectiveness and sustainability. The methodology advances sustainable synthetic practices and holds promise for scalable applications in medicinal and industrial chemistry. This work highlights the transformative potential of magnetic nanocatalysts in developing eco-conscious routes to biologically relevant heterocycles.

## Graphical Abstract

**Figure:**
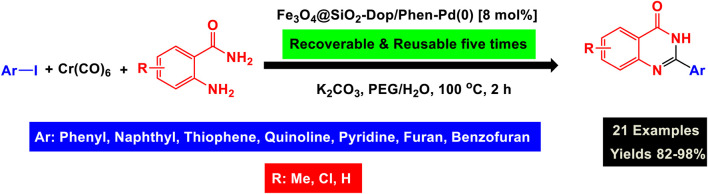

## Introduction

Quinazolinone compounds are important in medicinal chemistry due to having a benzene nucleus attached to pyrimidinone rings (Angulwar et al., 2022; Yu et al., 2018). These compounds have attracted attention as a large class of molecules showing many biological activities and luminescent properties (Moshkina et al., 2022). Medicinal activities include antimicrobial, antimalarial, anticonvulsant, anticancer, anti-inflammatory, and many other properties (Qiu et al., 2017). These compounds have a pharmacophoric structure in many biologically active natural, synthetic, pharmaceutical, agricultural, and veterinary products (Singh, 2023; Zhao et al., 2013). Several quinazolinone derivatives have shown promising anticancer activity by targeting various molecular pathways in tumor growth and proliferation (Hu et al., 2016; Mandal, 2023). Quinazolinones have anti-inflammatory effects, making them potential candidates for treating inflammatory diseases (Hu et al., 2016). Also, some quinazolinone derivatives have antioxidant properties, which could be beneficial in preventing oxidative stress-related diseases (Chortani et al., 2024). Therefore, due to their high diversity of pharmacological and biological activities, quinazolinones are considered one of the primary scaffolds in pharmaceutical research and play an important role in developing new drugs with diverse applications (Mahdavi et al., 2016).

Traditionally, quinazolinone synthesis involves using strong acids or bases and organic solvents, which harm human health and the environment (Borah et al., 2022). While these methods can offer high yields, they lack the flexibility and eco-friendliness required for broad adoption in green chemistry paradigms. Innovations to mitigate these downsides have included using water or other green solvents and biodegradable catalysts (Das et al., 2019). However, these approaches often face challenges such as lower reaction efficiency and the need for stringent reaction conditions. Research into green methods for synthesizing quinazolinones through carbonylative reactions is paramount, given these compounds’ significant role in medicinal chemistry (Yan et al., 2019). Traditional synthesis routes often involve harsh conditions and toxic reagents, raising serious environmental concerns. Green chemistry addresses these issues by minimizing environmental impact by using less hazardous materials, reducing waste, and enhancing overall efficiency (Peng et al., 2021). Furthermore, developing innovative synthetic pathways that utilize multicomponent reactions and eco-friendly solvents can lead to more sustainable and environmentally responsible production of quinazolinones (Breslow, 2010). By prioritizing green synthesis approaches, researchers can significantly contribute to advancing sustainable practices within the chemical industry. This promotes a safer and cleaner environment and ensures the continued availability of essential compounds for health and disease management (Odinets and Matveeva, 2010).

Palladium is a highly valuable catalyst for carbonylation reactions due to its ability to directly synthesize carbonyl compounds from simple and readily available feedstocks like carbon monoxide (Biglari and Jafarzadeh, 2024; Bontemps et al., 2011). This process exemplifies the principles of “atom economy” and “green chemistry,” as it efficiently incorporates CO into complex molecules without generating excessive waste (Dey et al., 2019). The versatility of palladium-catalyzed carbonylation is evident in its widespread application in the synthesis of pharmaceuticals, agrochemicals, and natural products, significantly advancing the field of organic synthesis (Hu and Kazemi, 2024; Kim et al., 2018). The advantages of palladium include its ability to catalyze reactions under milder conditions than other metals and its diverse range of reactions, such as aminocarbonylation, alkoxycarbonylation, and oxidative carbonylation (Gadge and Bhanage, 2014). These reactions offer efficient alternatives to traditional synthetic routes, often resulting in higher yields and fewer by-products (Barnard, 2008).

Magnetic catalysts have significantly advanced the field of chemical reactions, offering remarkable properties and compelling advantages that are hard to ignore (Rossi et al., 2014). These catalysts are particularly valued for their high surface area-to-volume ratio, which significantly enhances their catalytic activity, allowing for more efficient chemical transformations (Sead et al., 2025a; Yi et al., 2006). One of the primary advantages of using magnetic catalysts is their ease of separation from reaction mixtures by applying an external magnetic field, which streamlines the recovery process and minimizes waste (Govan and Gun’ko, 2014). This feature also contributes to the sustainability of chemical processes by enabling the reuse of catalysts, thereby minimizing material loss and environmental impact (Hou and Kazemi, 2024; Sead et al., 2025b; Zhang and Jialon, 2023). Additionally, magnetic catalysts can be directly heated through electromagnetic induction, offering a more energy-efficient and targeted heating method that avoids heating the entire reactor (Ahmad et al., 2025; Mokhtari and Naseri, 2024). This can lead to safer and cleaner reaction conditions and improved reaction kinetics (Kalantari et al., 2023; Kazemi et al., 2025). In industrial applications, magnetic catalysts synthesize important chemicals, pharmaceuticals, and fine chemicals with high yield and selectivity, showcasing their versatility and importance in advancing green chemistry practices (Dadashi et al., 2022; Shafik, 2023).

This study introduces an innovative approach to quinazolinone synthesis that aligns with green chemistry principles by employing a magnetically recoverable palladium catalyst. The catalyst, Fe_3_O_4_@SiO_2_-Dop/Phen-Pd (0), leverages the robust activity of palladium while facilitating easy recovery and reuse through its magnetic properties. This method utilizes a benign solvent system of polyethylene glycol (PEG) mixed with water and potassium carbonate as a base, which enhances the reaction’s eco-friendliness without compromising efficiency. This innovative approach holds great promise for the future of quinazolinone synthesis, offering a more sustainable and responsible method.

The catalyst is designed to engage in the carbonylative coupling of aryls or heteroaryl iodides with a carbonyl source and 2-aminobenzamide. In simpler terms, this means that the catalyst facilitates the joining of these compounds under significantly milder conditions than traditional methods. This novel catalyst system reduces the environmental impact by minimizing hazardous waste and demonstrates high catalytic activity with product yields ranging from 82% to 98% across various substrates.

The use of Fe_3_O_4_@SiO_2_-Dop/Phen-Pd (0) offers multiple advantages:

- High Yield and Efficiency: The catalyst performs impressively across a range of substrates, achieving high product yields in a shorter time frame. Its efficiency is a testament to the potential of innovative green synthesis methods in revolutionizing the chemical industry.
- Recyclability: The catalyst’s magnetic properties allow for straightforward separation using an external magnet, significantly reducing the waste and cost associated with catalyst recovery. This feature reassures the audience about the sustainability of the process, contributing to a cleaner and safer environment.
- Reduction in Toxicity: PEG/water as a reaction medium provides a non-toxic environment for the reactions and enhances the solubility of polar substrates, thereby reducing the need for organic solvents.

This study addresses the environmental concerns associated with traditional quinazolinone synthesis by integrating advanced catalytic materials with green chemistry principles. It opens new avenues for the sustainable production of medicinally valuable compounds. Future studies will focus on further optimizing the reaction conditions, expanding the range of substrates, and scaling up the process, ensuring that the practices are viable on an industrial scale. The continued development of such innovative methodologies promises to revolutionize the synthesis of complex molecules, supporting the pursuit of more sustainable and responsible chemical production strategies.

## Result and discussion

In this study, we present an innovative and efficient magnetic catalyst, palladium nanocomposite [Fe_3_O_4_@SiO_2_-Dop/Phen-Pd (0)], for synthesizing 2-aryl quinazolin-4(3*H*)-one derivatives. This synthesis employs carbonylation and cyclization reactions using aryl or heteroaryl iodides, a carbonyl source, and 2-aminobenzamide, with K_2_CO_3_ in a PEG/H_2_O medium. Our method significantly enhances reaction efficiency and yield, highlighting the catalyst’s potential in organic synthesis.

### Construction of Fe_3_O_4_@SiO_2_-Dop/Phen-Pd (0) catalyst

**SCHEME 1:**
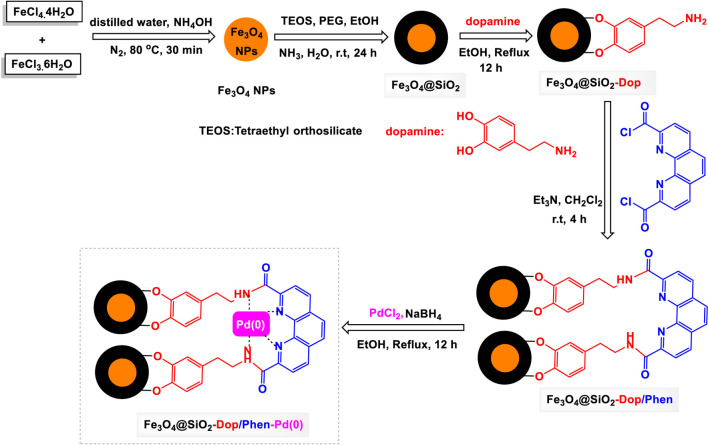
General method for the preparation of Fe_3_O_4_@SiO_2_-Dop/Phen-Pd (0) catalyst.

The Fe_3_O_4_@SiO_2_-Dop/Phen-Pd (0) catalyst was prepared using a simple method, according to the details in Scheme 1. First, Fe_3_O_4_ NPs were coated with silica, then their surface was modified with dopamine. Then, as a result of the ammonolysis of 1,10-phenanthroline-2,9-dicarbonyl dichloride by Fe_3_O_4_@SiO_2_-Dop nanocomposite, an attractive and practical magnetic ligand [Fe_3_O_4_@SiO_2_-Dop/Phen] was constructed. Then, by stabilizing the palladium complex in the presence of NaBH_4_ on the surface of the magnetic ligand, the desired palladium catalyst [Fe_3_O_4_@SiO_2_-Dop/Phen-Pd (0)] was successfully made, and its structure was analyzed by FT-IR, EDX, SEM, TEM, TGA, VSM, BET, AAS was studied.

**FIGURE 1:**
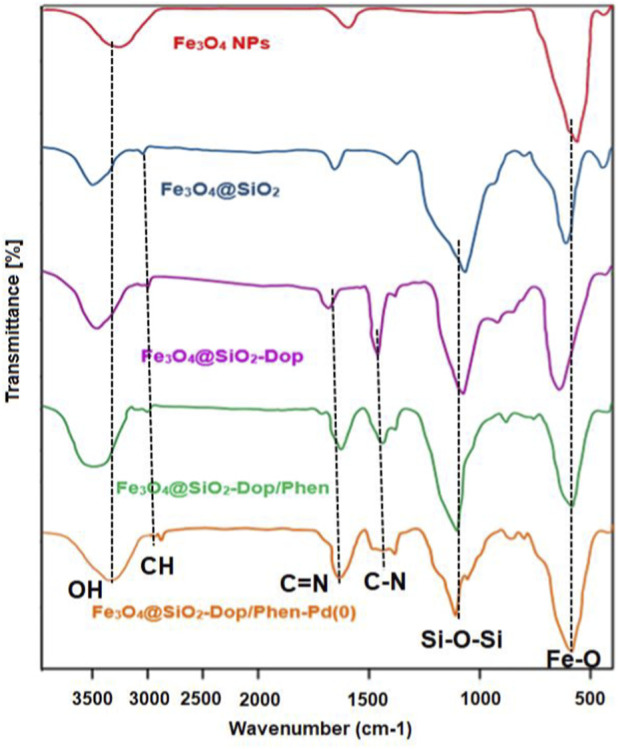
FT-IR spectrums of Fe_3_O_4_ NPs, Fe_3_O_4_@SiO_2_, Fe_3_O_4_@SiO_2_-Dop, Fe_3_O_4_@SiO_2_-Dop/Phen, and Fe_3_O_4_@SiO_2_-Dop/Phen-Pd (0) nanocomposites.

Figure 1 illustrates the Fourier Transform Infrared (FT-IR) spectra of various iron oxide nanocomposites, specifically Fe_2_O_3_ nanoparticles (NPs), Fe_2_O_3_@SiO_2_, Fe_2_O_3_@SiO_2_-Dop, Fe_2_O_3_@SiO_2_-Dop/Phen, and Fe_2_O_3_@SiO_2_-Dop/Phen-Pd (0). Each spectrum provides insight into the functional groups present in the materials and their structural characteristics.

1. Fe_2_O_3_ NPs Spectrum: The spectrum for Fe_2_O_3_ NPs (red line) shows characteristic absorption bands around 550 cm^-1^, which is attributed to the Fe-O bond stretching vibrations. This confirms the presence of iron oxide in its nanoparticle form.
2. Fe_2_O_3_@SiO_2_ Spectrum: Adding silica (blue line) results in new peaks between 1,000 cm^-1^ and 1,200 cm^-1^, corresponding to Si-O-Si stretching vibrations. This indicates the successful incorporation of SiO_2_ into the composite.
3. Fe_2_O_3_@SiO_2_-Dop Spectrum: The spectrum for Fe_2_O_3_@SiO_2_-Dop (green line) displays a slight shift and broadening of peaks compared to the previous sample, suggesting that doping has altered the electronic environment around both iron and silicon species.
4. Fe_2_O_3_@SiO_2_-Dop/Phen Spectrum: In this spectrum (orange line), additional peaks appear, indicating the presence of phenolic compounds due to doping. The peaks around 1,600 cm^-1^ may correspond to C=C stretching vibrations from aromatic rings, confirming phenol incorporation.
5. Fe_2_O_3_@SiO_2_-Dop/Phen-Pd (0) Spectrum: The final spectrum (brown line) shows further modifications with additional bands that can be attributed to palladium species. Notably, a peak around 1,500 cm^-1^ may indicate Pd-O interactions or metal coordination with organic ligands.

When comparing these spectra, several observations can be made regarding structural changes and functional group interactions:

- Functional Group Identification: Each subsequent modification from pure iron oxide NPs to more complex composites introduce new functional groups, as evidenced by shifting and emerging peaks in FT-IR spectra. This suggests successful synthesis and functionalization at each stage.
- Doping Effects: The transition from Fe_2_O_3_@SiO_2_ to Fe_2_O_3_@SiO_2_-Dop highlights how doping alters the material’s chemical environment, potentially enhancing catalytic properties or stability through electronic modifications.
- Role of Phenolic Compounds: Introducing phenolic compounds enhances interactions within the composite structure, as indicated by distinct spectral features in the Fe_2_O_3_@SiO_2_-Dop/Phen spectrum. This could suggest improved dispersion or stabilization of iron oxide nanoparticles within silica matrices.
- Palladium Incorporation: The final composite containing Pd (0) shows significant changes in spectral characteristics, indicating the successful incorporation of palladium into the matrix. This is critical for applications where palladium is a catalyst or promoter in various chemical reactions.

The FT-IR analysis presented in Figure 1 reveals significant insights into iron oxide nanocomposites’ structural and compositional evolution through various synthesis stages. Each modification introduces new functionalities and enhances potential applications in catalysis and materials science.

**FIGURE 2:**
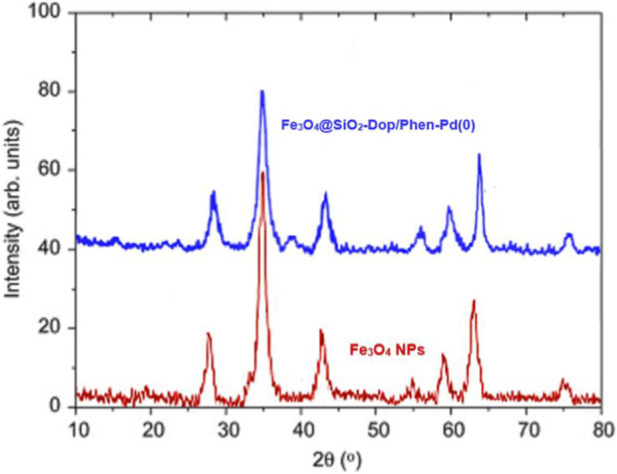
XRD patterns of Fe_3_O_4_ NPs, and Fe_3_O_4_@SiO_2_-Dop/Phen-Pd (0) catalyst.

Figure 2 displays the X-ray diffraction (XRD) patterns of Fe_3_O_4_ nanoparticles (NPs) and the composite catalyst Fe_3_O_4_@SiO_2_-Dop/Phen-Pd (0). The patterns provide insight into the material’s crystalline structure and phase purity.

1. Fe_3_O_4_ NPs (Red Pattern): The XRD pattern for Fe_3_O_4_ NPs exhibits distinct peaks at 30.1°, 35.5°, 43.2°, 53.6°, and 62.5° (2θ), corresponding to the (220), (311), (400), (422), and (511) planes, respectively. These peaks confirm the presence of a cubic spinel structure typical of magnetite, indicating good crystallinity.
2. Fe_3_O_4_@SiO_2_-Dop/Phen-Pd (0) Catalyst (Blue Pattern): The XRD pattern for the composite catalyst shows similar peaks to those of pure Fe_3_O_4_, confirming that the iron oxide phase is retained in the composite structure. However, there are additional broadening and slight shifts in peak positions, suggesting interactions between Fe_3_O_4_ and SiO_2_ and the incorporation of dopants and palladium.
- Crystallinity: Both patterns indicate high crystallinity for Fe_3_O_4_; however, the broader peaks in the composite suggest a reduction in crystallite size or increased disorder due to doping and functionalization with organic compounds and palladium.

Phase Integrity: The retention of characteristic peaks for Fe_3_O_4_ in both samples indicates that the composite’s synthesis process did not lead to significant phase transformation or degradation of iron oxide.

- Doping Effects: The introduction of SiO_2_ and other dopants modifies the structural characteristics slightly, which could enhance catalytic performance by increasing surface area or modifying electronic properties.
- Potential Catalytic Implications: The presence of palladium in the composite may impart unique catalytic properties not observed in pure Fe_3_O_4_ NPs alone, making this composite potentially more effective for specific reactions.

The XRD analysis presented in Figure 2 confirms that both pure Fe_3_O_4_ nanoparticles and the modified Fe_3_O_4_@SiO_2_-Dop/Phen-Pd (0) catalyst maintain their crystalline integrity while exhibiting variations indicative of structural modifications due to doping and functionalization. These findings suggest that such modifications could enhance catalytic activity, warranting further investigation into their practical applications in catalysis.

**FIGURE 3:**
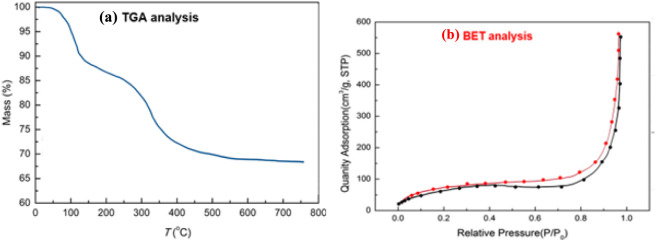
TGA **(a)** and BET **(b)** analyses of Fe_3_O_4_@SiO_2_-Dop/Phen-Pd (0) catalyst.

Figure 3 presents the Thermogravimetric Analysis (TGA) and Brunauer–Emmett–Teller (BET) analysis of the Fe_3_O_4_@SiO_2_-Dop/Phen-Pd (0) catalyst, providing insights into its thermal stability and surface area characteristics.

The left panel of Figure 3 shows the TGA curve, which indicates a gradual mass loss with increasing temperature. Key observations include:

- Initial Stability: The mass remains relatively stable up to approximately 200 °C, suggesting that the catalyst possesses good thermal stability in this range.
- Significant Mass Loss: A notable decrease in mass occurs between 200 °C and 400 °C, indicating the decomposition of organic components, likely from dopants or phenolic compounds incorporated into the structure.
- Final Residue: Beyond 400 °C, the mass stabilizes around 60%, suggesting that a significant portion of the organic material has been lost, while a stable inorganic framework remains.

The right panel of Figure 3 displays the BET adsorption isotherm for the Fe_3_O_4_@SiO_2_-Dop/Phen-Pd (0) catalyst:

- Surface Area: The BET analysis reveals a significant increase in adsorption quantity at low relative pressures (P/P_0_ < 0.5), indicating a high surface area and porosity of the catalyst.
- Type IV Isotherm: The isotherm shape suggests mesoporous characteristics, which are advantageous for catalytic applications as they facilitate reactant access to active sites.
- Maximum Adsorption: At P/P_0_ approaching 1.0, there is an asymptotic approach to saturation, confirming that the material can accommodate a substantial amount of gas.
- Thermal Stability vs. Surface Properties: The TGA results indicate that while the catalyst is thermally stable at lower temperatures, it undergoes significant changes upon heating due to organic component decomposition. In contrast, the BET analysis highlights its high surface area and porosity, essential for enhancing catalytic activity.
- Implications for Catalytic Activity: The combination of good thermal stability (as indicated by TGA) and high surface area (as shown by BET) suggests that this catalyst is well-suited for various catalytic processes. Pd (0) may further enhance its reactivity due to palladium’s known catalytic properties.

Based on the IUPAC classification system, this sample displays a type III isotherm curve, which indicates a specific interaction between the nitrogen gas and the material’s surface. The Brunauer-Emmett-Teller (BET) analysis of the Fe_3_O_4_@SiO_2_-Dop/Phen-Pd (0) catalyst reveals a surface area of 21.7 m^2^/g, suggesting a relatively modest capacity for gas adsorption. Additionally, the catalyst features an average pore diameter of 11.76 nm, reflecting its porous structure and potential for various catalytic applications.

The TGA and BET analyses in Figure 3 demonstrate that the Fe_3_O_4_@SiO_2_-Dop/Phen-Pd (0) catalyst exhibits favorable thermal stability and significant surface area characteristics.

**FIGURE 4:**
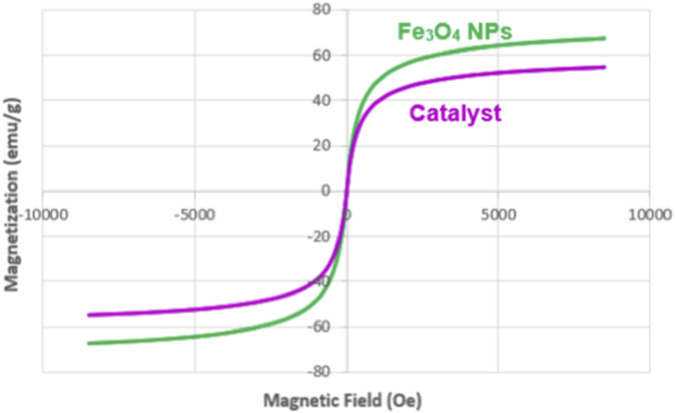
VSM spectrums of Fe_3_O_4_ NPs, and Fe_3_O_4_@SiO_2_-Dop/Phen-Pd (0) catalyst.

As depicted in Figure 4, the synthesis of Fe3O4 nanoparticles resulted in exceptional magnetic properties, measuring an impressive 68.347 emu/g. However, the introduction of surface modifications with silica, dopamine, and phenanthroline led to a noticeable reduction in the magnetic capabilities of the resulting nanocomposite. In a significant advancement, we stabilized the palladium complex, ultimately creating the Fe_3_O_4_@SiO_2_-Dop/Phen-Pd (0) catalyst. This synthesized catalyst showcases a commendable magnetic property of 53.196 emu/g, underscoring its remarkable effectiveness and potential for various applications.

**FIGURE 5:**
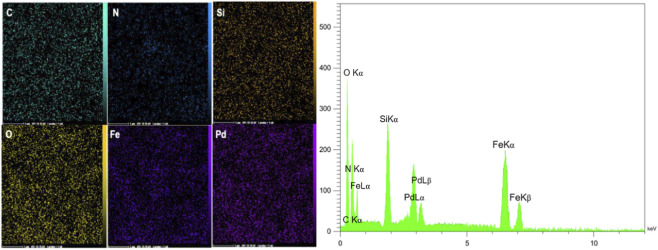
Elemental mapping and EDX analyses of the Fe_3_O_4_@SiO_2_-Dop/Phen-Pd (0) catalyst.

As seen in Figure 5, EDX and elemental mapping analysis confirmed that C, N, Si, O, Fe, and Pd elements are present in the nanocomposite structure, which can be a confirmation of the successful fabrication of the Fe_3_O_4_@SiO_2_-Dop/Phen-Pd (0) catalyst. Also, AAS analysis showed approximately 14.20 × 10^−5^ mol/g of palladium in the structure nanocomposite. ICP-OES analysis was done for more detailed analysis and confirmed that the amount of Pd in the catalyst equals 14.27 × 10^−5^ mol/g.

**FIGURE 6:**
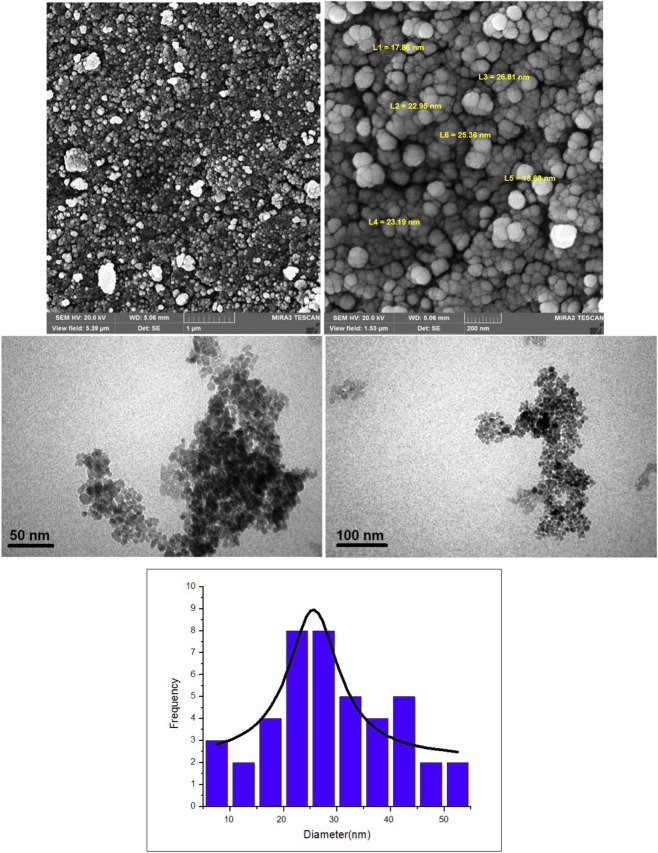
SEM and TEM images, and histogram distribution of Fe_3_O_4_@SiO_2_-Dop/Phen-Pd (0) catalyst.

The SEM images in the top row provide a detailed morphological analysis of the Fe_3_O_4_@SiO_2_-Dop/Phen-Pd (0) catalyst (Figure 6). The left SEM image shows a broad view of the catalyst’s surface, revealing a uniform distribution of particles with varying sizes. The right SEM image, taken at higher magnification, highlights individual spherical nanoparticles with size measurements labeled, ranging from approximately 17.5 nm–29.3 nm. This confirms the presence of well-dispersed nanoparticles and indicates a relatively uniform size distribution. The spherical morphology suggests practical synthesis and coating of the Fe_3_O_4_ core with the SiO_2_ and Pd (0) components. The TEM images in the middle row offer further insight into the catalyst’s nanoscale structure, showing agglomerated nanoparticles with high contrast, indicating the presence of metallic components. The scale bars (50 and 100 nm) confirm that the nanoparticles are within the nanometer range (Figure 6). The bottom histogram presents the particle size distribution, where most particles fall within the 20–30 nm range, with a peak frequency around 25 nm (Figure 6). The fitted curve suggests a Gaussian distribution, indicating a relatively controlled synthesis. This size distribution is crucial as it affects the catalyst’s surface area and reactivity, which is essential for catalytic applications.

### Catalytic investigation

**SCHEME 2:**
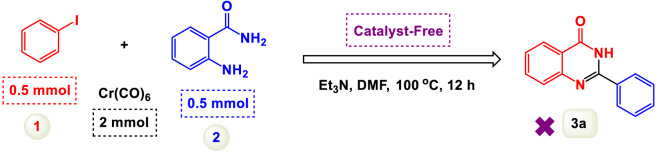
Performance of the model reaction under catalyst-free conditions.

We initially explored the reaction between 2-aminobenzamide and aryl iodide in the presence of carbon monoxide, using this as a crucial model reaction (Table 1). Conducted without a catalyst and with triethylamine (Et3N) as the base in dimethylformamide (DMF), this experiment aimed to assess the feasibility of the reaction. Despite our expectations, the results demonstrated that the model reaction did not succeed under these conditions, as detailed in Scheme 2. The reaction was conducted with the addition of a palladium catalyst, which facilitated the transformation into the desired product. To optimize the reaction conditions, we systematically tested different quantities of the catalyst. This approach enabled us to identify the most effective amount needed to achieve the best results for the synthesis. Increasing the amount of catalyst increased the efficiency of the co-catalyst system, and finally, the optimal amount of catalyst chosen for the reaction was determined to be 8 mol%. Experiments revealed that increasing the catalyst concentration beyond this level did not significantly enhance the reaction’s progress, suggesting that 8 mol% is the effective threshold for achieving desired results.

**TABLE 1:** Effect of Fe_3_O_4_@SiO_2_-Dop/Phen-Pd (0) catalyst on the model reaction (product 3a).

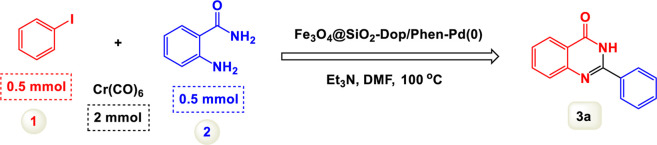			
Entry	Catalyst (mol%)	Time (h)	Yield (%) ^a^
1	5	4	76%
2	6	4	82%
3	7	4	86%
4	8	3	88%
5	9	3	88%
6	10	3	88%

^a^ Yields referred to isolated products.

Then, to select the base, the reaction was thoroughly assessed with various bases to explore their effectiveness. Incorporating a base played a pivotal role in facilitating the reaction (Table 2), as the formation of the desired product was impossible in its absence. After conducting a series of tests with different bases, it became clear that K_2_CO_3_ stood out for its exceptional efficiency, proving to be the most effective option for achieving optimal results in the reaction. By understanding the optimal amount of catalyst and base, the effect of the solvent on the model reaction was evaluated in the next step. The results of optimizing the solvent for the preparation of quinazolines also confirmed that the choice of solvent is essential for system efficiency, significantly affecting reaction rates and component stability. The results showed that toluene, THF, and dioxane solvents are unsuitable for the reaction. The best results were observed when water, DMF, and PEG solvents were used as solvents. However, the highest efficiency was seen when the mixture of PEG and water (2:1) was used as a solvent.

**TABLE 2:** Effect of base and solvent on the model reaction (product 3a).

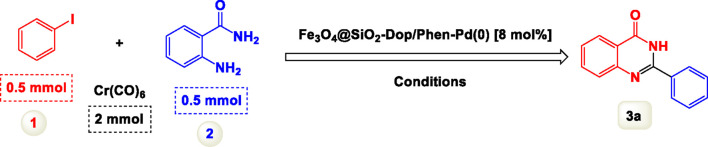				
Entry	Base	Solvent (^o^C)	Time (h)	Yield (%) ^a^
1	Et_3_N	DMF (100 °C)	3	88%
2	DBU	DMF (100 °C)	3	61%
3	K_2_CO_3_	DMF (100 °C)	3	92%
4	KOAc	DMF (100 °C)	3	90%
5	K_3_PO_4_	DMF (100 °C)	3	43%
6	KOH	DMF (100 °C)	3	65%
7	Cs_2_CO_3_	DMF (100 °C)	3	84%
8	NaOH	DMF (100 °C)	3	52%
9	^*t*^-BuOK	DMF (100 °C)	3	13%
10	No	DMF (100 °C)	3	No
11	K_2_CO_3_	Solvent-Free (100 °C)	12	5%
12	K_2_CO_3_	EtOH (Reflux)	3	91%
13	K_2_CO_3_	Water (Reflux)	2	95%
14	K_2_CO_3_	Anisole (100 °C)	4	90%
15	K_2_CO_3_	Toluene (100 °C)	6	51%
16	K_2_CO_3_	THF (Reflux)	6	38%
17	K_2_CO_3_	DMSO (100 °C)	4	89%
18	K_2_CO_3_	MeCN (Reflux)	3	90%
19	K_2_CO_3_	Dioxane (100 °C)	4	65%
20	K_2_CO_3_	PEG (100 °C)	2	96%
21	K_2_CO_3_	PEG/H_2_O (1/1) (100 °C)	2	96%
22	K_2_CO_3_	PEG/H_2_O (2/1) (100 °C)	2	98%
23	K_2_CO_3_	PEG/H_2_O (1/2) (100 °C)	2	96%
24	K_2_CO_3_	PEG/H_2_O (2/1) (90 °C)	2	90%
25	K_2_CO_3_	PEG/H_2_O (2/1) (110 °C)	2	98%
26	K_2_CO_3_	PEG/H_2_O (2/1) (120 °C)	2	98%

^a^ Yields referred to isolated products.

**SCHEME 3:**
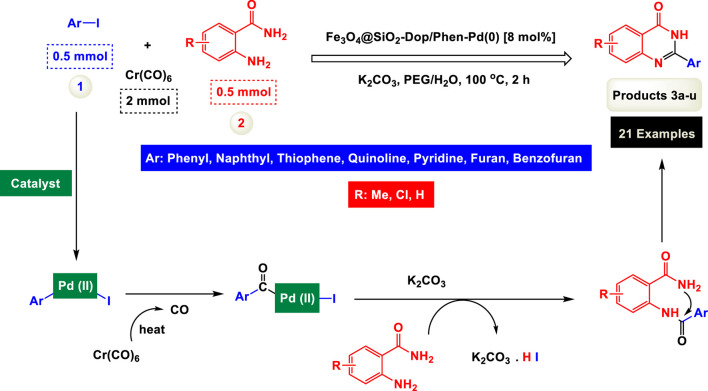
Scope of Fe_3_O_4_@SiO_2_-Dop/Phen-Pd (0) catalyst for synthesis of 2-aryl quinazolin-4(3*H*)-ones.

By identifying the optimal conditions,^a^we can significantly enhance our understanding and, in the next stage, effectively explore the breadth of the catalytic system to create a variety of valuable quinazoline derivatives through the reaction of 2-aminobenzamides with different derivatives of aryl iodides in the presence of a carbonyl source (Scheme 3). These experiments’ findings demonstrated the catalytic system’s remarkable efficiency. Notably, all products were synthesized with high and acceptable yields, as detailed in Table 3. This underscores the system’s effectiveness and highlights its potential for applications requiring reliable and productive synthesis processes. The synthesis of the products demonstrated significantly higher yields when the substrates contained an electron-donating group attached to the aromatic ring. This indicates that the presence of such groups enhances the efficiency of the reaction, leading to a more favorable outcome in the production process. Also, the results were very satisfactory when heteroaryl iodide derivatives were used. The physical characteristics of the synthesized products were fully aligned with those of the previously documented samples, demonstrating consistency in their properties. Furthermore, the structural integrity of these compounds was confirmed through detailed analyses using 1H NMR and 13C NMR spectroscopy.

**TABLE 3:** Scope of Fe_3_O_4_@SiO_2_-Dop/Phen-Pd (0) catalyst for synthesis of 2-aryl quinazolin-4(3*H*)-ones.

Entry	Product (3a-u)	Yield (%)^a^	Melting point [Reference]
1	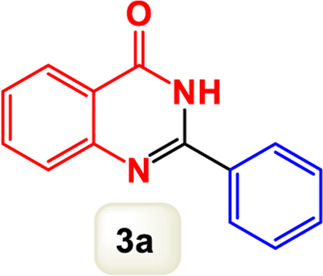	98%	235 °C–237 °C (Zhao et al., 2013)
2	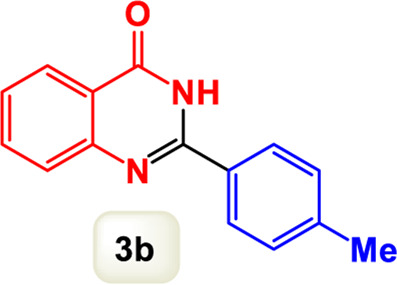	^a^6%	243 °C–245 °C (Das et al., 2019)
3	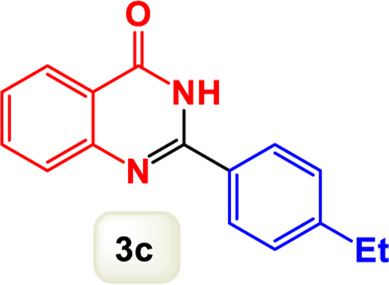	96%	204 °C–206 °C (Che et al., 2023)
4	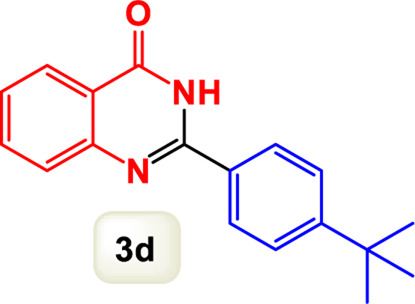	89%	224 °C–226 °C (Chortani et al., 2024)
5	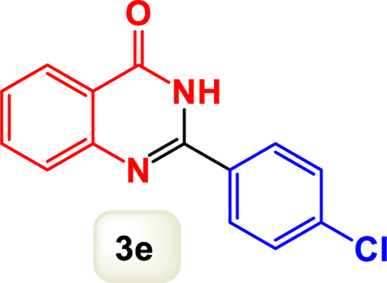	83%	297 °C–299 °C (Qiu et al., 2017)
6	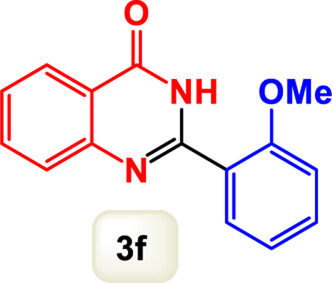	92%	209 °C–211 °C (Mahdavi et al., 2016)
7	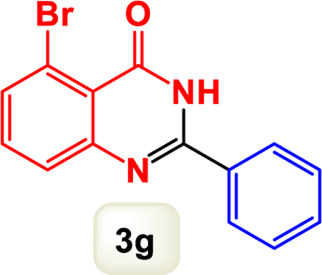	86%	283 °C–285 °C (Zhao et al., 2016)
8	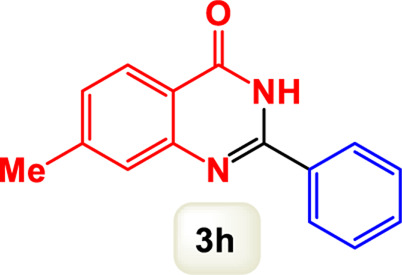	93%	245 °C–247 °C (Liu et al., 2024)
9	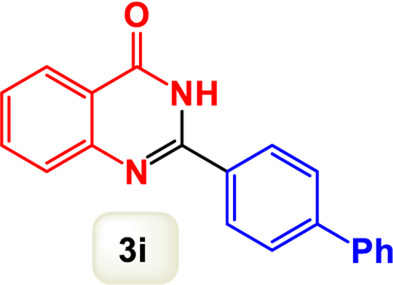	94%	287 °C–289 °C (Huang et al., 2024)
10	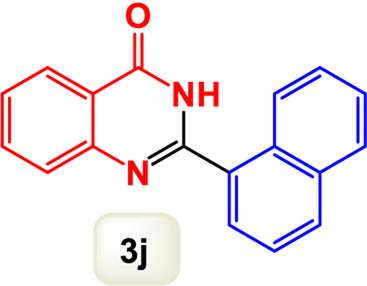	97%	281 °C–283 °C (Yan et al., 2019)
11	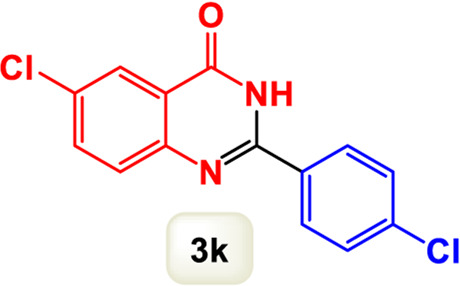	82%	298 °C–300 °C (Potewar et al., 2005)
12	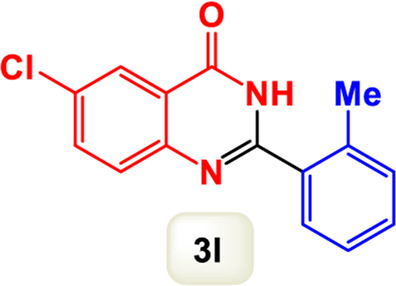	88%	287 °C–289 °C (Peng et al., 2021)
13	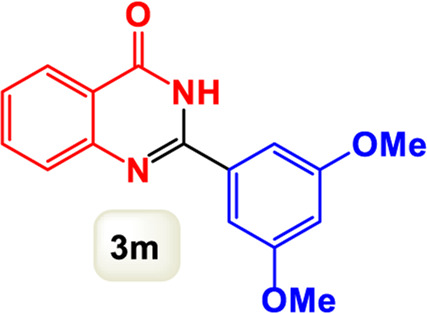	90%	280 °C–282 °C (Sharma et al., 2016)
14	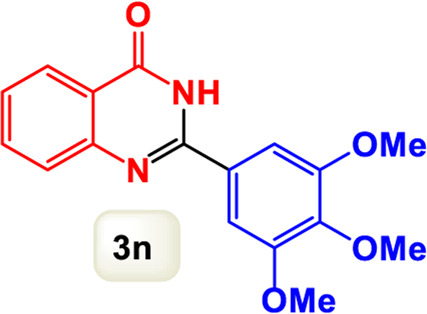	91%	258 °C–260 °C (Matcha et al., 2021)
15	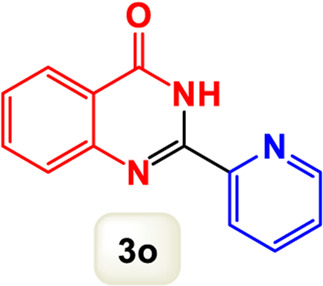	98%	171 °C–173 °C (Hu et al., 2016)
16	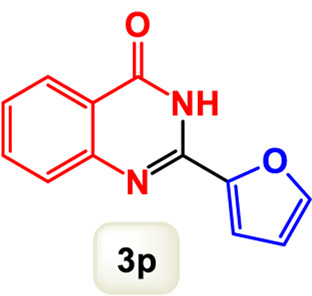	91%	233 °C–235 °C (Feng et al., 2023)
17	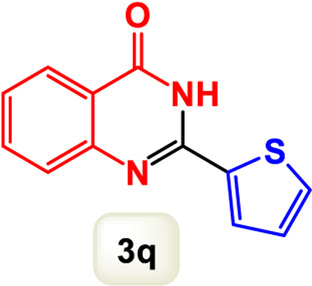	93%	271 °C–173 °C (Małecki et al., 2024)
18	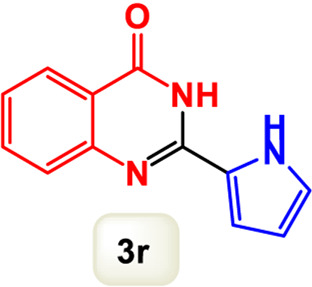	94%	271 °C–173 °C (Borah et al., 2022)
19	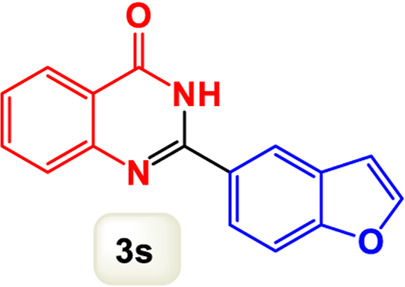	93%	280 °C–282 °C (Biswas et al., 2024)
20	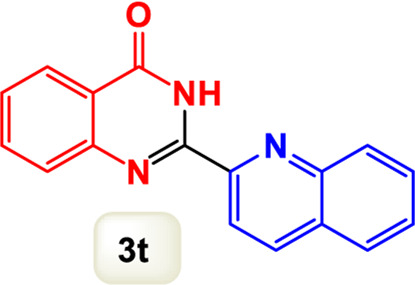	96%	227 °C–229 °C (Moshkina et al., 2022)
21	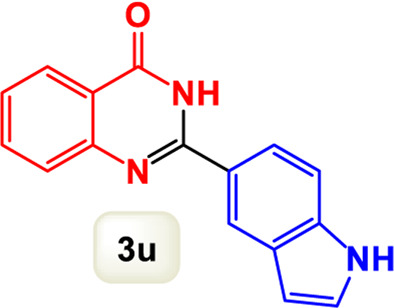	95%	Oil (Biswas et al., 2024)

^a^ Yields referred to isolated products.

The mechanism illustrated in Scheme 3 outlines the synthesis of 2-aryl quinazolin-4(3*H*)-ones using a palladium-catalyzed reaction involving aryl iodides and an amine. The process begins with activating the palladium catalyst, specifically Fe_3_O_4_@SiO_2_-Dop-Phen-Pd (0), crucial in facilitating the coupling reaction.

Initially, an aryl iodide (Ar—I) is combined with Cr(CO)_6_ and an amine (R-NH_2_) under specific conditions. Chromium hexacarbonyl’s presence generates carbon monoxide and activates the palladium species. The reaction proceeds through forming a palladium (II) complex, where the aryl iodide undergoes oxidative addition to the palladium center, forming a Pd(II) intermediate.

Subsequently, this Pd(II) complex interacts with carbon monoxide and heat, leading to a further transformation that allows for the introduction of the amine moiety. During this step, the amine reacts with another Pd(II) species, yielding a new intermediate containing both the aryl group and the amine functionality.

The next phase involves deprotonation facilitated by potassium carbonate (K_2_CO_3_), which acts as a base to promote nucleophilic attack on the carbonyl carbon. This step is critical as it forms a key intermediate yielding the final product after tautomerization. The reaction is conducted in a solvent mixture of polyethylene glycol (PEG) and water at elevated temperatures (100 °C) for 2 hours, which enhances solubility and promotes efficient interaction between reactants.

The catalyst’s role throughout this mechanism is pivotal; it accelerates each step and stabilizes various intermediates, thus improving overall yields. The use of Fe_3_O_4_@SiO_2_-Dop-Phen-Pd (0) ensures that palladium remains active while also providing magnetic properties that facilitate easy separation from the reaction mixture post-reaction. This characteristic makes recycling of the catalyst feasible, contributing to economic and environmental sustainability.

Ultimately, this catalytic system enables the synthesis of diverse 2-aryl quinazolin-4(3*H*)-ones from various substrates, showcasing its versatility across different aryl groups (such as phenyl, naphthyl, thiophene, etc.) and functional groups (like methyl and chlorine). The successful generation of 21 distinct products highlights the effectiveness of this catalytic approach and its potential applications in organic synthesis.

In the field of catalyst science, evaluating the catalyst’s reusability is crucial from the perspective of green chemistry. The Fe_3_O_4_@SiO_2_-Dop/Phen-Pd (0) catalyst reusability was investigated by synthesizing model product 3a under standardized conditions. To recover the Fe_3_O_4_@SiO_2_-Dop/Phen-Pd (0) catalyst after use, first, allow the reaction mixture to settle with a strong magnet to attract the catalyst. Carefully decant the supernatant liquid, then wash the catalyst with ethanol or deionized water 2–3 times to remove residual reactants. Dry the washed catalyst at ≤60 °C in an oven or in a vacuum desiccator overnight. Finally, store the dried catalyst in labeled glass vials or airtight containers for future use.

**FIGURE 7:**
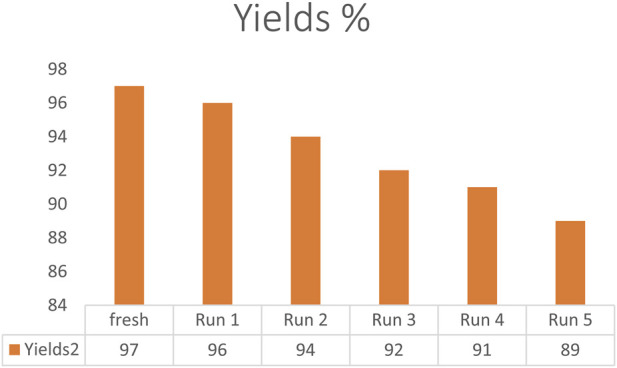
Reusability of Fe_3_O_4_@SiO_2_-Dop/Phen-Pd (0) catalyst in synthesis of product 3a.

**FIGURE 8:**
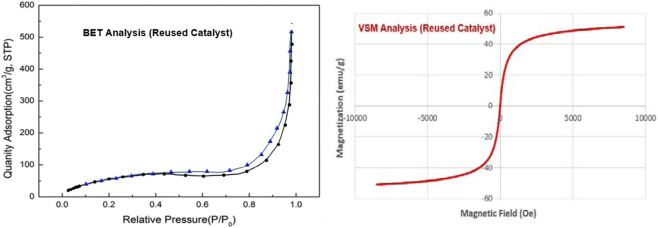
BET and VSM analyses of the reused Fe_3_O_4_@SiO_2_-Dop/Phen-Pd (0) catalyst (after 5 times).

**FIGURE 9:**
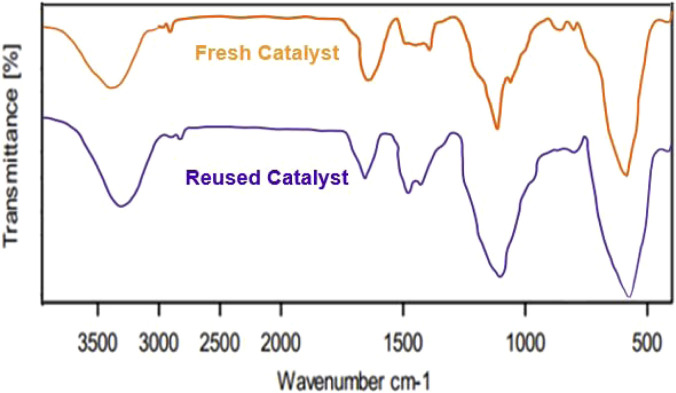
FT-IR spectrums of the fresh and reused Fe_3_O_4_@SiO_2_-Dop/Phen-Pd (0) catalyst (after 5 times).

The findings from the recovery tests provided compelling evidence that the Fe_3_O_4_@SiO_2_-Dop/Phen-Pd (0) catalyst maintains its effectiveness even after being utilized in numerous reactions. Specifically, it showcased robust catalytic performance across five cycles, demonstrating only a minimal decline in activity, as illustrated in Figure 7. FT-IR and XRD analyses demonstrated that the recovered catalyst remains stable and well-preserved, even after being reused five times (Figures 8, 9). The VSM analysis illustrated in Figure 8 indicates that the reused catalyst retains a significant magnetic property, measured at 50.763 emu/g. In addition, the ICP-OES analysis revealed that the reused catalyst contains approximately 14.24 × 10×-5 mol/g of palladium embedded within its structure. This finding suggests a slight decrease in palladium concentration compared to the catalyst’s initial state, indicating some loss during reuse.

The results of the synthesis of 2-aryl quinazolin-4(3*H*)-ones in this method were compared with previously reported methods. As you can see in Table 4, This approach offers numerous key advantages over reported methods such as: using a magnetic catalyst with easy separation and high reusability, performing carbonylation reactions in only 2 h, synthesizing the products with efficiency above, performing the reaction in green solvent, applicability many substrates such as aryl and heteroaryl iodides.

**TABLE 4:** Comparison of the efficiency of this method with other methods for 2-aryl quinazolinones.

Entry	Product (Fe_3_O_4_@SiO_2_-Dop/Phen-Pd (0) K_2_CO_3_, PEG/H2O, 100 °C, 2 h)	Catalyst	Conditions	Yield (%) [Ref]
1	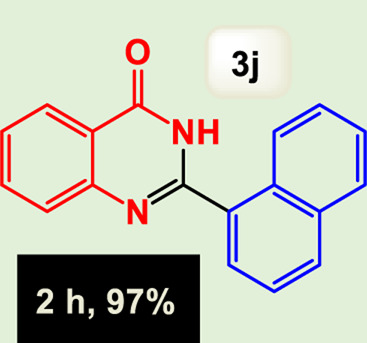	Pd(OAc)_2_/BuPAd_2_	DBU, DMF, 120 °C, 16 h	77% (Wu et al., 2013)
2	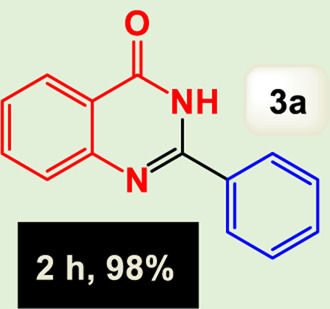	Cu(OAc)_2_	BuOK, BuOH, 100 °C, 16 h	80% (Yu et al., 2018)
3	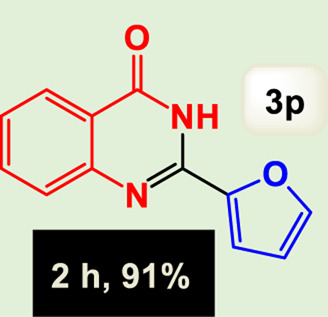	CuI/TMSN_3_	DMSO, Air, 80 °C, 24 h	78% (Upadhyaya et al., 2016)
4	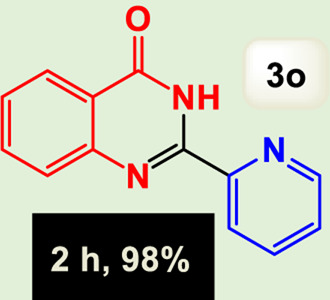	Cu(II)/Azo Ligand	Toluene, NaOH, 85 °C, 16 h	74% (Das et al., 2019)
5	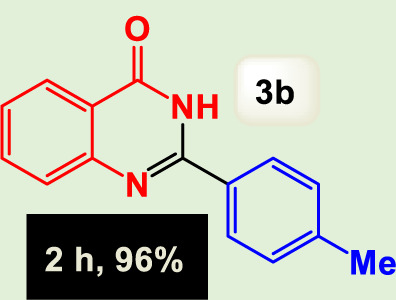	Iodine	DMSO, Air, 110 °C, 16 h	78% (Mohammed et al., 2015)

## Conclusion

This study introduces an innovative magnetic catalyst, Fe_3_O_4_@SiO_2_-Dop/Phen-Pd (0), for synthesizing 2-aryl quinazolin-4(3H)-one derivative via carbonylation and cyclization reactions. Utilizing aryl or heteroaryl iodides, a carbonyl source, and 2-aminobenzamides, the reaction employs an eco-friendly medium of polyethylene glycol (PEG) mixed with water, facilitated by potassium carbonate. The hybrid nanocomposite structure of the catalyst, which incorporates magnetic Fe_3_O_4_ nanoparticles, SiO_2_, and palladium complexed with dopamine and phenanthroline ligands, is designed to enhance stability and catalytic activity. This design principle and the catalyst’s magnetic properties simplify catalyst recovery.

The catalyst’s successful synthesis and functional integration are well-documented and characterized extensively by techniques such as FT-IR, TGA, EDX, and XRD. The catalyst displays exceptional reusability and maintains high efficiency over multiple cycles, presenting a significant advantage in cost-effectiveness and environmental sustainability. However, it is important to note that the catalyst may have limitations in certain reaction conditions or with specific substrates. The catalyst proves effective across various substrates, with reactions completed in just 2 hours, yielding superior results.

Emphasizing green chemistry, using non-toxic PEG/water as a solvent aligns with efforts to reduce hazardous waste without compromising performance. The versatility of the Fe_3_O_4_@SiO_2_-Dop/Phen-Pd (0) catalyst offers potential extensions beyond quinazolinone synthesis to include other vital heterocyclic compounds. This study enhances the methodology of synthesizing biologically active molecules and sets the stage for broader applications in sustainable and cost-effective chemical production. Future research could focus on optimizing the catalyst’s performance, exploring its applicability in other reactions, or scaling up its production for industrial use.

## Data Availability

The original contributions presented in the study are included in the article/Supplementary Material, further inquiries can be directed to the corresponding author.
