# Supplementary material for: Sustainable synthesis of quinazolinones: exploring multicomponent reactions with a novel magnetic palladium catalyst
Source: Front Chem. 2026 Apr 22;14:1632736. doi: 10.3389/fchem.2026.1632736 (PMC13143982; doi:10.3389/fchem.2026.1632736)

**Sustainable Synthesis of Quinazolinones: Exploring Multicomponent Reactions with a Novel Magnetic Palladium Catalyst**

Xiaotong Liang ^1^*, Ziqi Yang ^1^, Bo Li ^1^

^1^ Yangling Vocational & Technical College, Yangling, Shaanxi, 712100, China

*** Corresponding author**: [xl7943184@gmail.com](mailto:xl7943184@gmail.com)

Ziqi Yang: [yang-work@foxmail.com](mailto:yang-work@foxmail.com)

Bo Li: [18710802302@163.com](mailto:18710802302@163.com)

**The reusability of the Fe_3_O_4_@SiO_2_-Dop/Phen-Pd(0) catalyst**

In the field of catalyst science, evaluating the catalyst's reusability is crucial from the perspective of green chemistry. The Fe_3_O_4_@SiO_2_-Dop/Phen-Pd(0) catalyst reusability was investigated by synthesizing model product 3a under standardized conditions. To recover the Fe₃O₄@SiO₂-Dop/Phen-Pd(0) catalyst after use, first, allow the reaction mixture to settle with a strong magnet to attract the catalyst. Carefully decant the supernatant liquid, then wash the catalyst with ethanol or deionized water 2-3 times to remove residual reactants. Dry the washed catalyst at ≤60°C in an oven or in a vacuum desiccator overnight. Finally, store the dried catalyst in labeled glass vials or airtight containers for future use.

The findings from the recovery tests provided compelling evidence that the Fe_3_O_4_@SiO_2_-Dop/Phen-Pd(0) catalyst maintains its effectiveness even after being utilized in numerous reactions. Specifically, it showcased robust catalytic performance across five cycles, demonstrating only a minimal decline in activity, as illustrated in Figure 7. FT-IR and XRD analyses demonstrated that the recovered catalyst remains stable and well-preserved, even after being reused five times (Figures 8 and 9). The VSM analysis illustrated in Figure 8 indicates that the reused catalyst retains a significant magnetic property, measured at 50.763 emu/g. In addition, the ICP-OES analysis revealed that the reused catalyst contains approximately 14.24×10×-5 mol/g of palladium embedded within its structure. This finding suggests a slight decrease in palladium concentration compared to the catalyst's initial state, indicating some loss during reuse.

**Figure 7. Reusability of Fe_3_O_4_@SiO_2_-Dop/Phen-Pd(0) catalyst in synthesis of product 3a.**

**
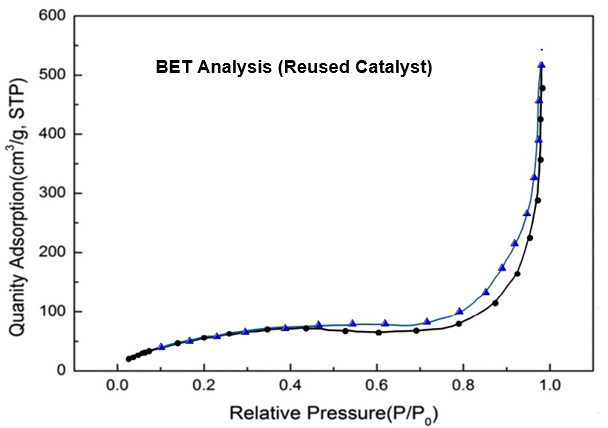

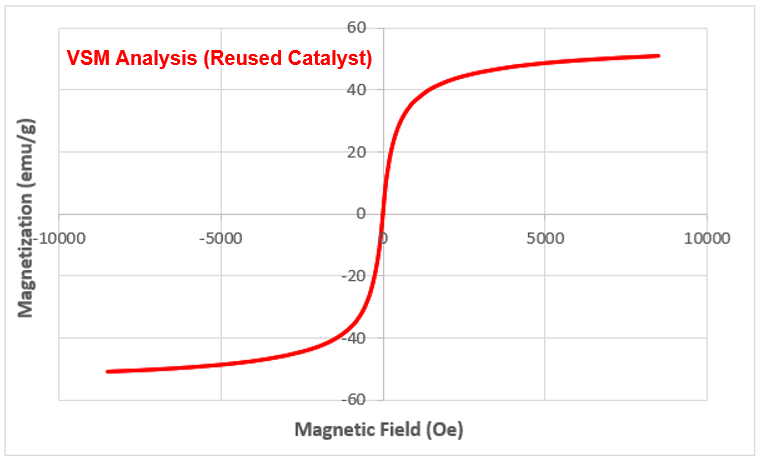
**

**Figure 8. BET and VSM analyses of the reused Fe_3_O_4_@SiO_2_-Dop/Phen-Pd(0) catalyst (after 5 times).**

**
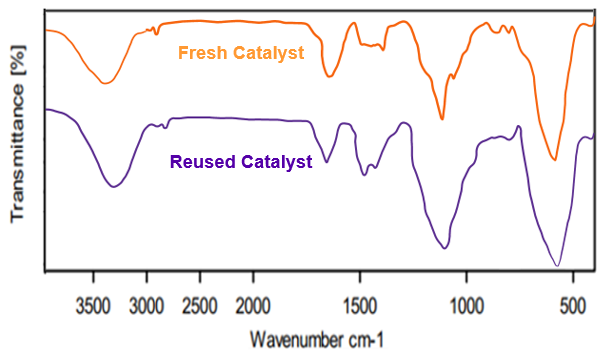
**

**Figure 9. FT-IR spectrums of the fresh and reused Fe_3_O_4_@SiO_2_-Dop/Phen-Pd(0) catalyst (after 5 times).**

**Experimental**

**Materials and methods**

The study utilized chemicals that were sourced from commercial suppliers and were used without any additional purification. The NMR spectra were acquired using a 400 MHz spectrometer at a temperature of 25°C in CDCl_3_.

Fourier-Transform Infrared (FT-IR) spectroscopy was performed using a KBr pellet method with an Alpha Bruker FT-IR spectrophotometer, covering the spectral range of 400–4000 cm⁻¹. The crystallinity of the nanomaterials was analyzed using an X-ray diffraction (XRD) instrument (Philips Xpert) with Cu-Kα radiation (λ = 0.1545 nm). To study surface morphology and elemental composition, a MIRA 3-XMU field emission scanning electron microscope (FE-SEM) equipped with energy dispersive X-ray spectroscopy (EDX) was employed. Thermogravimetric analysis (TGA) was conducted on a Mettler TA4000 system under a nitrogen atmosphere, with samples heated from 25°C to 800°C at a rate of 15°C/min. The magnetic properties of the nanoparticles were characterized using a vibrating sample magnetometer (VSM, Model 730) at room temperature.

**Preparation of Fe_3_O_4_ NPs**

In a controlled nitrogen atmosphere, a mixture of 5.838 grams of iron(III) chloride hexahydrate and 2.147 grams of iron(II) chloride tetrahydrate was dissolved in 100 mL of deionized water. Ammonia (10 mL) was added over 30 minutes while stirring, resulting in a black dispersion. The mixture was then heated to 80°C for 30 minutes, followed by magnetic decantation to separate the precipitate. The black solid was washed with double-distilled water until neutral, then with ethanol, and dried at room temperature for further analysis or application.

**Preparation of the Fe_3_O_4_@SiO_2_**

The synthesized Fe_3_O_4_ NPs weighing 2 grams were meticulously dispersed in 20 mL of water using sonication for a duration of 30 minutes, ensuring a uniform suspension. Following this, 200 mL of 2-propanol was introduced into the reaction mixture, enhancing the system's overall dynamics. The mixture was then stirred continuously with a magnetic stirrer at room temperature to maintain an optimal reaction environment. As the stirring progressed, several key ingredients were systematically added to the suspension. First, 5.36 grams of polyethylene glycol (PEG) were incorporated, followed by an additional 20 mL of water. To this, 10 mL of a 28 wt.% ammonia solution was added, introducing a basic environment conducive to the next phase of the reaction. Finally, 2 mL of tetraethyl orthosilicate (TEOS) was introduced to the growing mixture, allowing for the formation of a silica coating. The reaction was sustained with continuous stirring for an impressive 38 hours at room temperature, ensuring the components thoroughly interacted and reacted. Once the product, Fe_3_O_4_@SiO_2_, was formed, it was carefully isolated using an external magnet. To ensure purity, the resulting compounds were washed twice with both ethanol and distilled water, removing any residual materials and leaving behind the desired nanocomposite.

**Preparation of Fe_3_O_4_@SiO_2_-Dop nanocomposite**

The synthesized Fe_3_O_4_@SiO_2_ powder, weighing 2 grams, was carefully dispersed in 40 milliliters of ethanol (EtOH) using an ultrasonic bath for 30 minutes, ensuring a homogenous mixture. Following this, 2 grams of 4-(2-aminoethyl)benzene-1,2-diol, commonly known as dopamine, were added to the reaction mixture. This combination was then stirred mechanically for a duration of 12 hours at room temperature, allowing for effective interaction between the components. After the required reaction time, the Fe_3_O_4_@SiO_2_-Dop nanocomposite was isolated by applying an external magnet, facilitating easy separation from the liquid. The composite was then thoroughly washed three times with deionized water and n-hexane to remove any unreacted materials. Finally, the washed nanocomposite was dried at room temperature under vacuum conditions to obtain a pure product.

**Preparation of Fe_3_O_4_@SiO_2_-Dop/Phen nanocomposite**

The Fe_3_O_4_@SiO_2_-Dop nanocomposite was synthesized by combining 2 grams of Fe_3_O_4_@SiO_2_-Dop with 3 grams of 1,10-phenanthroline-2,9-dicarbonyl dichloride and 2.5 mmol (0.252 g) of triethylamine (Et_3_N) in 50 mL of dichloromethane (CH_2_Cl_2_). The mixture underwent sonication for 30 minutes for proper dispersion and was then stirred at room temperature for 4 hours to promote the reaction. Finally, the obtained nanomaterial (Fe_3_O_4_@SiO_2_-Dop/Phen) was collected using an external magnet and dried under vacuum to remove residual solvent, resulting in a stable product ready for further applications.

**Preparation of Fe_3_O_4_@SiO_2_-Dop/Phen-Pd(0) nanocomposite**

To prepare the Fe_3_O_4_@SiO_2_-Dop/Phen-Pd(0) nanocomposite, we initiated the process by adding 5 mmol of PdCl_2_ to a well-mixed suspension of 1 gram of ultrasonically dispersed Fe_3_O_4_@SiO_2_-Dop/Phen in 50 mL of ethanol. This mixture was then refluxed for 12 hours, which facilitated the integration of palladium ions into the nanocomposite structure. After refluxing, sodium borohydride (NaBH_4_) was introduced in an amount of 2 mmol to reduce the Pd(II) ions to Pd(0), continuing within the ethanol medium. Upon completion of the synthesis, the Fe_3_O_4_@SiO_2_-Dop/Phen-Pd(0) nanocomposite was magnetically separated from the solution. It was subsequently washed thoroughly with hot water and ethanol to eliminate any residual materials. Finally, the product was dried at 80 °C, yielding a stable form of the desired nanocomposite.

**General approach for preparation of 2-aryl quinazolin-4(3*H*)-ones**

In a representative chemical reaction, a mixture comprising aryl iodides (0.5 mmol), Cr(CO)₆ (0.1 mmol), 2-aminobenzamides (0.5 mmol), Fe₃O₄@SiO₂-Dop/Phen-Pd(0) catalyst (8 mol%), and K₂CO₃ (1.5 equivalents) in a solvent system of PEG/H₂O (2:1, 3 mL) was stirred mechanically at 100°C for 2 hours. [1-3] The reaction progress was monitored by TLC, after which the catalyst was easily separated using an external magnetic field. The mixture was then diluted with 10 mL of dichloromethane (CH₂Cl₂), washed sequentially with 20 mL of 1 M HCl, and thrice with 10 mL of brine. The organic layer was dried over anhydrous Na₂SO₄, filtered, and concentrated under vacuum. The crude product was purified by flash chromatography using petroleum ether and ethyl acetate as eluents. The physical characteristics of the purified products matched reported data, and their structures were confirmed through ¹H NMR and ¹³C NMR analyses.

1. S. Bahadorikhalili, S. Ansari, H. Hamedifar, L. Ma'mani, M. Babaei, R. Eqra, M. Mahdavi, Mo (CO)6-assisted Pd-supported magnetic graphene oxide-catalyzed carbonylation-cyclization as an efficient way for the synthesis of 4(3H)-quinazolinones. 2019, 33, e4769. https://doi.org/10.1002/aoc.4769

2. M. Niakan, Z. Asadi, M. Emami, Binuclear Palladium Complex Immobilized on Mesoporous SBA-16: Efficient Heterogeneous Catalyst for the Carbonylative Suzuki Coupling Reaction of Aryl Iodides and Arylboronic Acids Using Cr(CO)_6_ as Carbonyl Source. *Catal Lett* 2020, 150, 404–418. https://doi.org/10.1007/s10562-019-03087-w

3. I. Ahmad, M. K. Abosaoda, Y. Jadeja, G. Sangwan, V. Kavitha, A. Kashyap, S. Homurotova, M. Kazemi and R. Javahershenas, A Comprehensive Review on Carbonylation Reactions: Catalysis by Magnetic Nanoparticles Supported Transition Metals. Nanoscale Adv., 2025, 7, 3189 – 3209. https://doi.org/ 10.1039/D5NA00040H.

**NMR data for 2-aryl quinazolin-4(3*H*)-ones**

**2-phenylquinazolin-4(3*H*)-one [98%]**

M.P: 235-237 ºC. ^1^H NMR (400 MHz, CDCl_3_) δ 11.16 (s, 1H), 8.12 (d, J = 8.4 Hz, 1H), 7.97 (t, J = 7.8 Hz, 1H), 7.66-7.60 (m ,2H), 7.33 (d, J = 7.9 Hz, 2H), 7.27-7.18 (m, 1H). ^13^C NMR (101 MHz, CDCl_3_) δ 169.0, 154.2, 147.5, 135.4, 133.7, 131.0, 129.8, 127.3, 126.2, 125.1, 123.6, 121.4.

**2-(p-tolyl)quinazolin-4(3*H*)-one [96%]**

M.P: 243-245 ºC. ^1^H NMR (400 MHz, CDCl_3_) δ 11.09 (s, 1H), 8.08 (d, J = 7.7 Hz, 2H), 7.95-7.90 (m, 2H), 7.45-7.40 (m ,2H), 7.38-7.27 (m, 2H), 2.45 (s, 3H). ^13^C NMR (101 MHz, CDCl_3_) δ 168.6, 154.3, 142.9, 134.2, 130.1, 129.6, 127.5, 126.4, 125.7, 123.3, 121.5, 21.8.

**2-(4-ethylphenyl)quinazolin-4(3*H*)-one [96%]**

M.P: 204-206 ºC. ^1^H NMR (400 MHz, CDCl_3_) δ 11.21 (s, 1H), 8.10 (d, J = 8.4 Hz, 1H), 7.96-7.91 (m, 1H), 7.65 (d, J = 9.0 Hz, 2H), 7.45 (d, J = 8.7 Hz, 2H), 7.36-7.30 (m, 2H),2.74-2.70 (m, 2H), 1.33 (t, J = 6.8 Hz, 3H). ^13^C NMR (101 MHz, CDCl_3_) δ 168.4, 154.2, 147.6, 134.1, 131.7, 128.9, 127.4, 126.5, 125.3, 123.7, 121.0, 28.5, 15.3.

**2-(4-(tert-butyl)phenyl)quinazolin-4(3H)-one [89%]**

M.P: 224-226 ºC. ^1^H NMR (400 MHz, CDCl_3_) δ 11.47 (s, 1H), 8.06 (d, J = 9.3 Hz, 1H), 7.77 (d, J = 7.9 Hz, 1H), 7.45 (t, J = 7.4 Hz, 1H), 7.36-7.30 (m, 1H), 7.24 (d, J = 9.6 Hz, 2H), 7.11 (d, J = 8.6 Hz, 2H), 1.43 (s, 9H). ^13^C NMR (101 MHz, CDCl_3_) δ 164.5, 153.2, 142.9, 136.3, 129.7, 128.3, 127.0, 126.5, 125.3, 124.4, 122.7, 119.0, 35.2, 31.9.

**2-(4-chlorophenyl)quinazolin-4(3*H*)-one [83%]**

M.P: 297-299 ºC. ^1^H NMR (400 MHz, CDCl_3_) δ 11.47 (s, 1H), 8.18 (d, J = 9.0 Hz, 1H), 7.89-7.84 (m, 1H), 7.65 (d, J = 7.7 Hz, 2H), 7.51 (d, J = 7.8 Hz, 2H), 7.43-7.31 (m, 2H).^13^C NMR (101 MHz, CDCl_3_) δ 162.0, 154.3, 148.1, 137.2, 135.9, 132.1, 129.5, 128.7, 126.1, 125.6, 124.0, 121.3.

**2-(2-methoxyphenyl)quinazolin-4(3*H*)-one [92%]**

M.P: 209-211 ºC. ^1^H NMR (400 MHz, CDCl_3_) δ 11.43 (s, 1H), 8.09 (d, J = 9.2 Hz, 1H), 7.87-7.82 (m, 2H), 7.46 (d, J = 7.8 Hz, 1H), 7.33 (t, J = 7.6 Hz, 2H), 7.03 (d, J = 7.8 Hz, 1H), 3.85 (s, 3H). ^13^C NMR (101 MHz, CDCl_3_) δ 161.4, 154.3, 137.8, 128.7, 127.3, 126.1, 125.9, 124.5, 121.0, 117.5, 105.4, 56.7.

**5-bromo-2-phenylquinazolin-4(3*H*)-one [86%]**

M.P: 283-285 ºC. ^1^H NMR (400 MHz, CDCl_3_) δ 11.39 (s, 1H), 8.63 (d, J = 9.1 Hz, 1H), 8.22 (d, J = 8.3 Hz, 1H), 8.04 (d, J = 7.8 Hz, 1H), 7.66-7.63 (m, 2H), 7.55-7.48 (m, 3H).^13^C NMR (101 MHz, CDCl_3_) δ 164.3, 153.9, 138.7, 136.8, 133.6, 131.0, 129.6, 128.4, 127.4, 126.8, 122.3.

**7-methyl-2-phenylquinazolin-4(3*H*)-one [93%]**

M.P: 245-247 ºC. ^1^H NMR (400 MHz, CDCl_3_) δ 11.37 (s, 1H), 8.10 (d, J = 8.6 Hz, 1H), 7.97 (d, J = 8.4 Hz, 1H), 7.85 (d, J = 7.7 Hz, 2H), 7.53 (s, 1H), 7.44-7.40 (m, 1H), 7.22 (d, J = 7.8 Hz, 2H), 2.48 (s, 3H). ^13^C NMR (101 MHz, CDCl_3_) δ 168.3, 154.8, 138.2, 135.4, 133.0, 131.7, 129.1, 128.6, 126.5, 125.3, 124.2, 123.9, 121.6, 21.3.

**2-([1,1'-biphenyl]-4-yl)quinazolin-4(3*H*)-one [94%]**

M.P: 287-289 ºC. ^1^H NMR (400 MHz, CDCl_3_) δ 11.43 (s, 1H), 8.16 (d, J = 8.7 Hz, 1H), 8.02 (d, J = 6.8 Hz, 1H), 7.92 (d, J = 8.0 Hz, 2H), 7.77 (d, J = 8.7 Hz, 2H), 7.62 (d, J = 8.4 Hz, 2H), 7.56-7.51 (m, 3H), 7.44-7.40 (m, 2H). ^13^C NMR (101 MHz, CDCl_3_) δ 167.4, 153.9, 142.7, 140.3, 136.2, 132.1, 129.7, 128.3, 127.9, 127.6, 126.8, 125.2, 124.6, 123.1, 121.0.

**2-(naphthalen-1-yl)quinazolin-4(3*H*)-one [97%]**

M.P: 281-283 ºC. ^1^H NMR (400 MHz, CDCl_3_) δ 11.55 (s, 1H), 8.59 (d, J = 9.0 Hz, 1H), 8.21 (d, J = 8.7 Hz, 1H), 8.17 (d, J = 6.8 Hz, 2H), 7.98 (d, J = 8.0 Hz, 2H), 7.86-7.80 (m, 1H), 7.56-7.51 (m, 3H), 7.48 (t, J = 7.7 Hz, 1H). ^13^C NMR (101 MHz, CDCl_3_) δ 167.5, 154.2, 136.7, 134.2, 133.9, 131.2, 129.6, 128.1, 127.4, 126.5, 125,6, 125.0, 124.3, 124.1, 123.8, 121.7.

**6-chloro-2-(4-chlorophenyl)quinazolin-4(3*H*)-one [82%]**

M.P: 298-300 ºC. ^1^H NMR (400 MHz, CDCl_3_) δ 11.60 (s, 1H), 8.17 (s, 1H), 8.09 (dd, J = 8.4, 4.3 Hz, 1H), 7.73 (d, J = 7.6 Hz, 2H), 7.53-7.50 (m, 1H), 7.45 (d, J = 7.9 Hz, 2H). ^13^C NMR (101 MHz, CDCl_3_) δ 162.0, 154.3, 148.1, 137.2, 135.9, 132.1, 129.5, 128.7, 126.1, 125.6, 124.0, 121.3.

**6-chloro-2-(o-tolyl)quinazolin-4(3*H*)-one [88%]**

M.P: 287-289 ºC. ^1^H NMR (400 MHz, CDCl_3_) δ 11.52 (s, 1H), 8.05 (dd, J = 10.1, 2.3 Hz, 1H), 7.98 (dd, J = 9.7, 3.1 Hz, 1H), 7.55 (s, 1H), 7.47-7.40 (m, 2H), 7.33-7.23 (m, 2H), 2.43 (s, 3H). ^13^C NMR (101 MHz, CDCl_3_) δ 162.0, 154.1, 137.6, 136.4, 129.7, 128.4, 127.1, 126.5, 125.4, 124.2, 122.0, 22.0.

**2-(3,5-dimethoxyphenyl)quinazolin-4(3*H*)-one [90%]**

M.P: 280-282 ºC. ^1^H NMR (400 MHz, CDCl_3_) δ 11.49 (s, 1H), 8.05 (d, J = 7.6 Hz, 1H), 7.77 (d, J = 7.4 Hz, 1H), 7.43-7.30 (m, 2H), 7.25 (s, 2H), 6.89 (s, 1H), 3.81 (s, 6H). ^13^C NMR (101 MHz, CDCl_3_) δ 165.1, 159.0, 153.2, 130.6, 129.8, 125.3, 124.8, 123.5, 122.1, 115.4, 56.7.

**2-(3,4,5-trimethoxyphenyl)quinazolin-4(3*H*)-one [91%]**

M.P: 258-260 ºC. ^1^H NMR (400 MHz, CDCl_3_) δ 11.65 (s, 1H), 7.94 (d, J = 7.3 Hz, 1H), 7.75 (d, J = 7.8 Hz, 1H), 7.38-7.35 (m, 1H), 7.17 (t, J=9.1 Hz, 1H), 6.47 (s, 2H), 3.98 (s, 6H), 3.75 (s, 3H). ^13^C NMR (101 MHz, CDCl_3_) δ 167.5, 165.3, 154.8, 139.7, 134.3, 131.7, 129.4, 128.9, 127.5, 126.1, 121.6, 104.3, 58.9, 52.7

**2-(pyridin-2-yl)quinazolin-4(3*H*)-one [98%]**

M.P: 171-173 ºC. ^1^H NMR (400 MHz, CDCl_3_) δ 11.52 (s, 1H), 8.56 (d, J = 8.5Hz, 1H), 8.19 (d, J = 7.7 Hz, 1H), 7.98 (d, J = 9.1 Hz, 1H), 7.74-7.70 (m, 1H), 7.58 (d, J = 7.6 Hz, 1H), 7.47-7.43 (m, 1H), 7.24 (d, J = 7.6 Hz, 2H). ^13^C NMR (101 MHz, CDCl_3_) δ 168.9, 155.2, 152.1, 148.7, 137.6, 135.2, 127.0, 126.1, 125.4, 124.3, 122.8, 121.9.

**2-(furan-2-yl)quinazolin-4(3*H*)-one [91%]**

M.P: 233-235 ºC. ^1^H NMR (400 MHz, CDCl_3_) δ 11.51 (s, 1H), 8.05 (d, J = 7.4 Hz, 1H), 7.84 (d, J = 9.1 Hz, 1H), 7.66 (d, J = 7.7 Hz, 1H), 7.45-7.40 (m, 2H), 7.37 (t, J = 7.9 Hz, 1H), 7.10 (t, J = 7.6 Hz, 1H). ^13^C NMR (101 MHz, CDCl_3_) δ 162.0, 154.1, 136.5, 134.9, 129.9, 128.7, 127.9, 126.4, 125.7, 124.3, 122.0.

**2-(thiophen-2-yl)quinazolin-4(3*H*)-one [93%]**

M.P: 271-173 ºC. ^1^H NMR (400 MHz, CDCl_3_) δ 11.55(s, 1H), 8.10-8.00 (m, 3H), 7.55-7.50 (m, 1H), 7.48 (d, J = 7.8 Hz, 2H), 7.27-7.20 (m, 1H). ^13^C NMR (101 MHz, CDCl_3_) δ 167.9, 154.0, 136.5, 135.8, 134.1, 130.5, 129.7, 128.0, 127.6, 122.0, 121.8.

**2-(1*H*-pyrrol-2-yl)quinazolin-4(3*H*)-one [94%]**

M.P: 271-173 ºC. ^1^H NMR (400 MHz, CDCl_3_) δ 12.27(s, 1H), 11.62 (s, 1H), 8.02 (d, J = 7.6 Hz, 1H), 7.88 (d, J = 8.4 Hz, 1H), 7.54 (d, J = 7.7 Hz, 2H), 7.48-7.41 (m, 1H), 7.26-7.23 (m, 2H). ^13^C NMR (101 MHz, CDCl_3_) δ 164.3, 153.1, 137.8, 136.5, 131.7, 130.6, 127.1, 125.3, 123.4, 122.0, 121.4.

**2-(benzofuran-5-yl)quinazolin-4(3*H*)-one [93%]**

M.P: 280-282 ºC. ^1^H NMR (400 MHz, CDCl_3_) δ 11.54 (s, 1H), 8.04 (d, J = 8.8 Hz, 1H), 7.96 (s, 1H), 7.76 (d, J = 8.4 Hz, 1H), 7.46-7.40 (m, 2H), 7.39-7.30 (m, 2H), 7.18-7.10 (m, 1H), 7.09 (dd, J=9.1, 3.2 Hz, 1H). ^13^C NMR (101 MHz, CDCl_3_) δ 164.9, 154.7, 139.1, 138.3, 137.6, 136.1, 133.4, 132.8, 131.7, 130.6, 129.7, 128.5, 127.3, 126.5, 125.0, 124.2, 122.5.

**2-(quinolin-2-yl)quinazolin-4(3*H*)-one [96%]**

M.P: 227-229 ºC. ^1^H NMR (400 MHz, CDCl_3_) δ 11.44 (s, 1H), 8.07 (d, J = 7.7 Hz, 1H), 7.83 (d, J = 7.8 Hz, 1H), 7.76 (d, J = 8.6 Hz, 2H), 7.47-7.41 (m, 2H), 7.33 (t, J = 7.6 Hz, 2H), 7.28-7.23 (m, 2H). ^13^C NMR (101 MHz, CDCl_3_) δ 164.8, 154.9, 133.0, 132.9, 131.2, 130.6, 129.1, 128.5, 127.6, 126.3, 125.0, 124.3, 123.1, 122.3.

**2-(1*H*-indol-5-yl)quinazolin-4(3*H*)-one [95%]**

Oil, ^1^H NMR (400 MHz, CDCl_3_) δ 11.65 (s, 1H), 10.23 (s, 1H), 9.12 (s, 1H), 8.09 (dd, J = 9.8, 3.3 Hz, 1H), 7.96 (d, J = 7.9 Hz, 1H), 7.54 (t, J = 8.6 Hz, 1H), 7.44-7.40 (m, 1H), 7.37-7.31 (m, 2H), 7.18 (dd, J = 7.8. 5.6 Hz, 1H), 6.28 (d, J = 8.6 Hz, 1H). ^13^C NMR (101 MHz, CDCl_3_) δ 171.9, 154.2, 138.9, 136.5, 134.8, 129.4, 128.1, 127.0, 125.7, 124.2, 123.1,122.6.


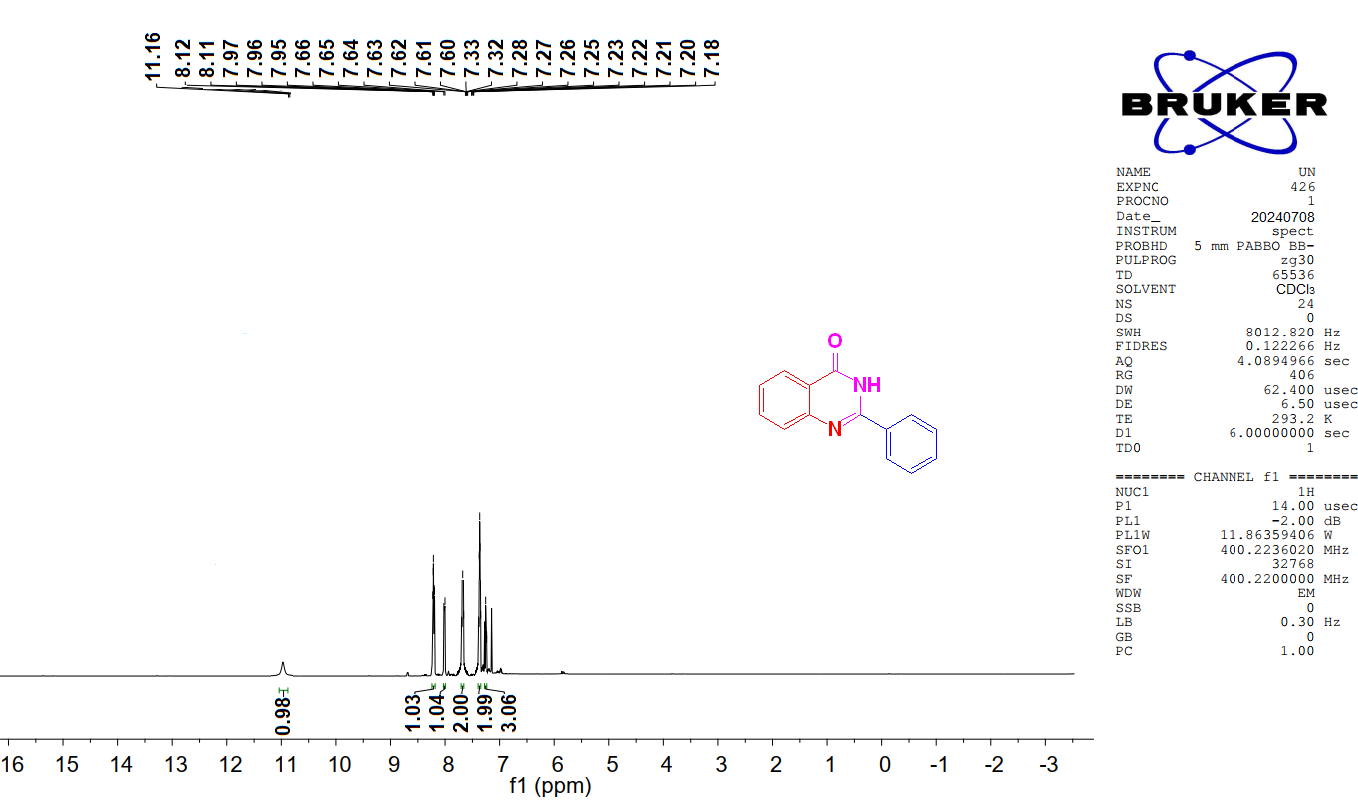


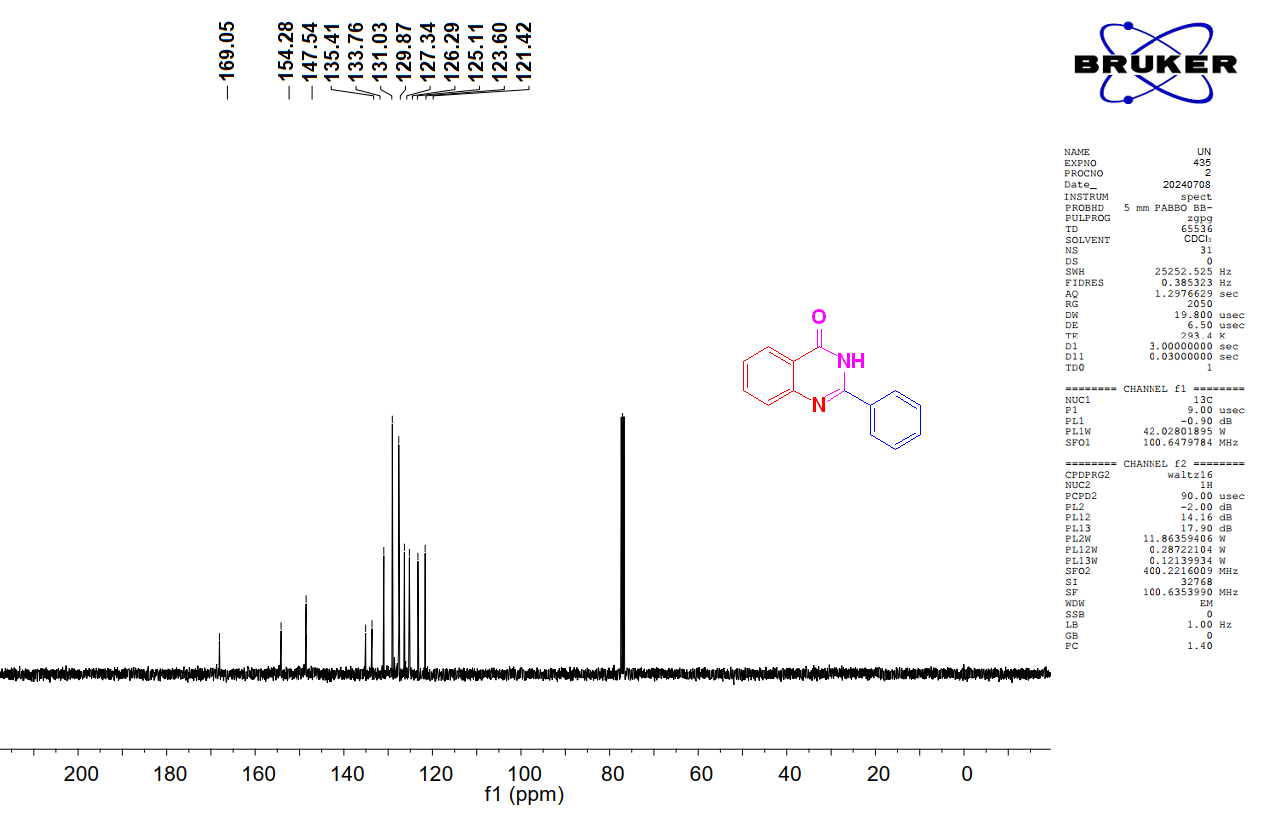


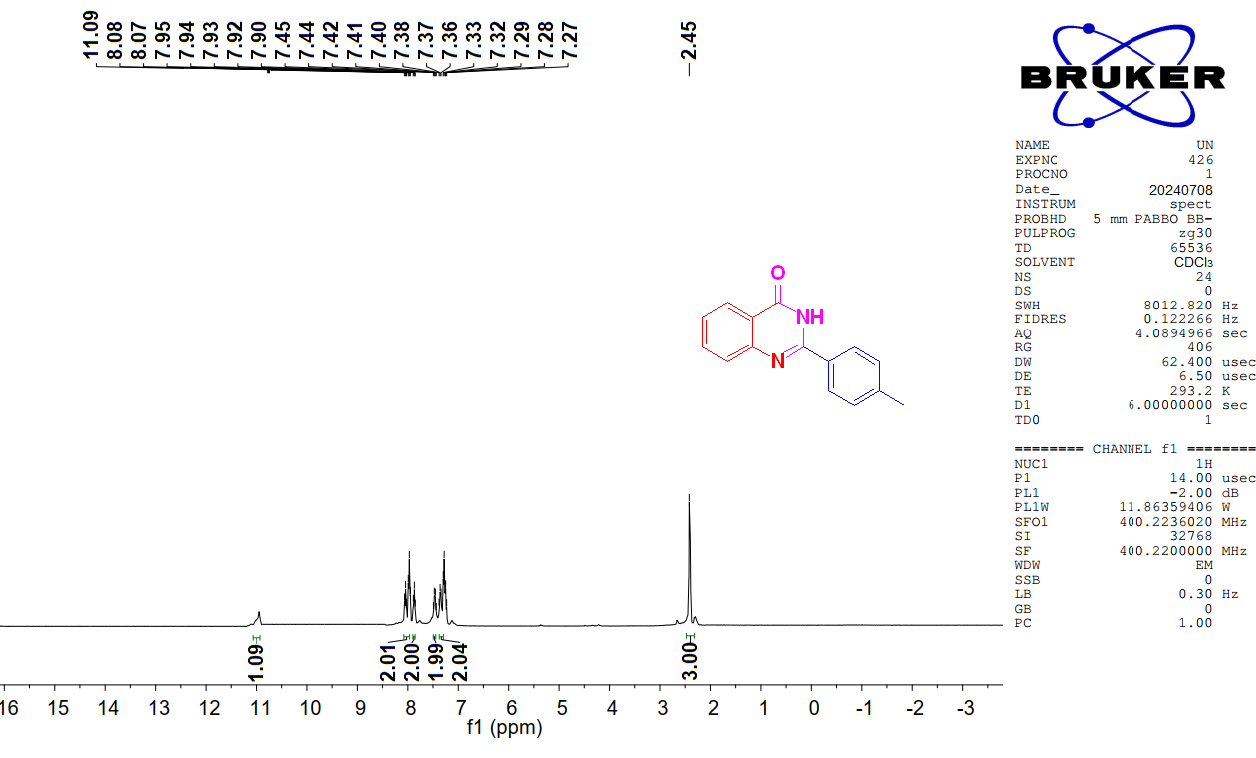


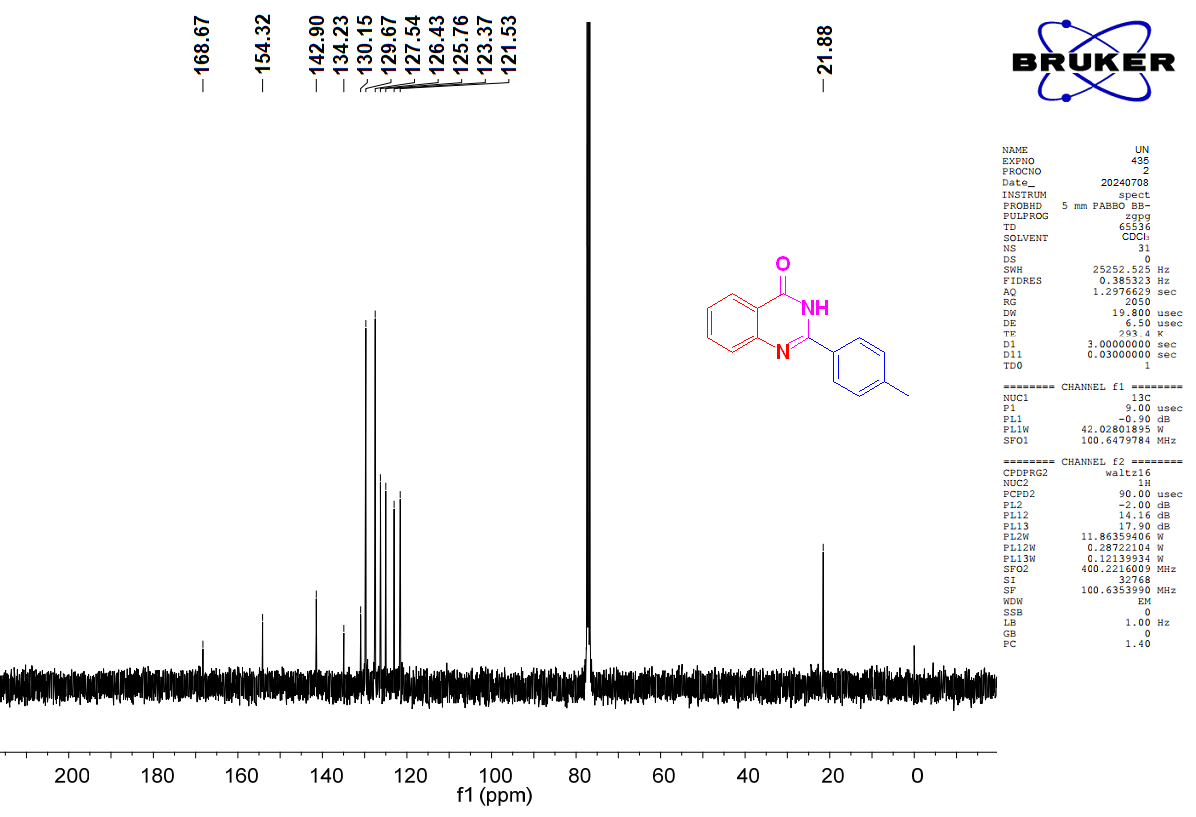


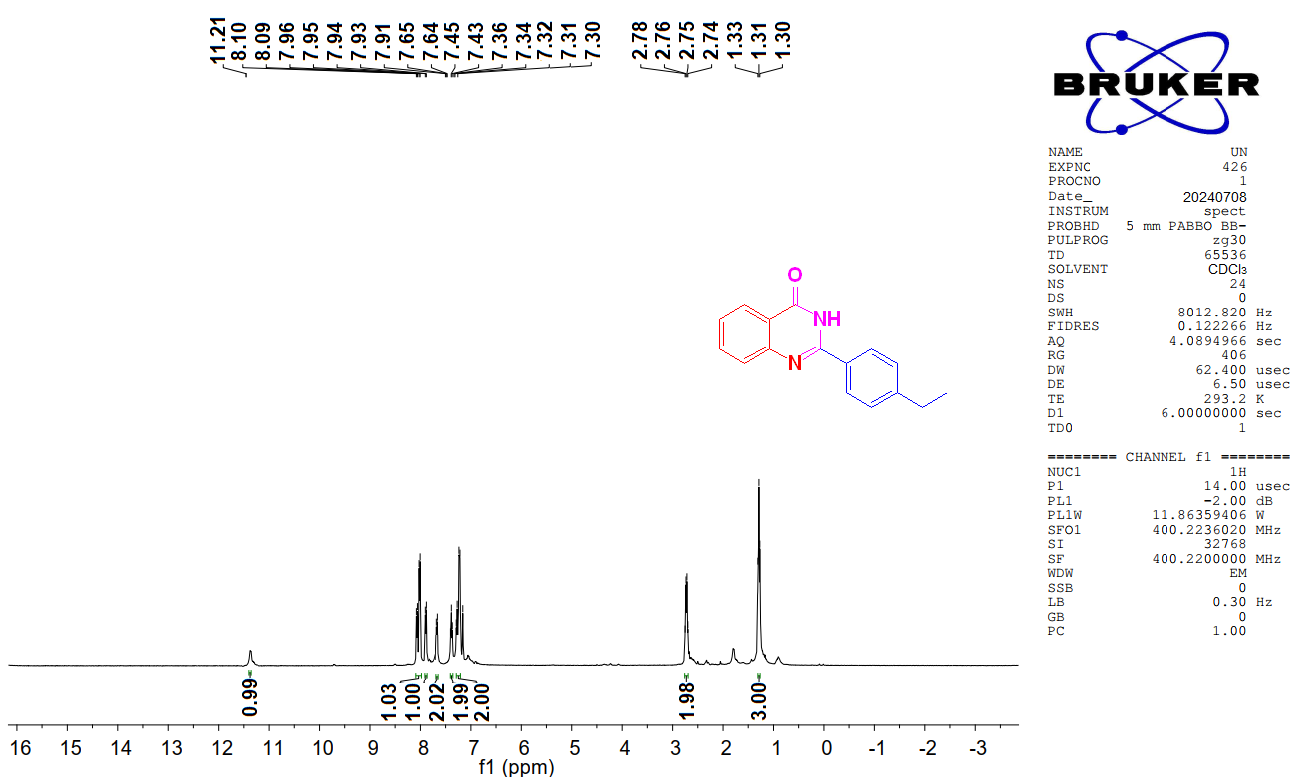


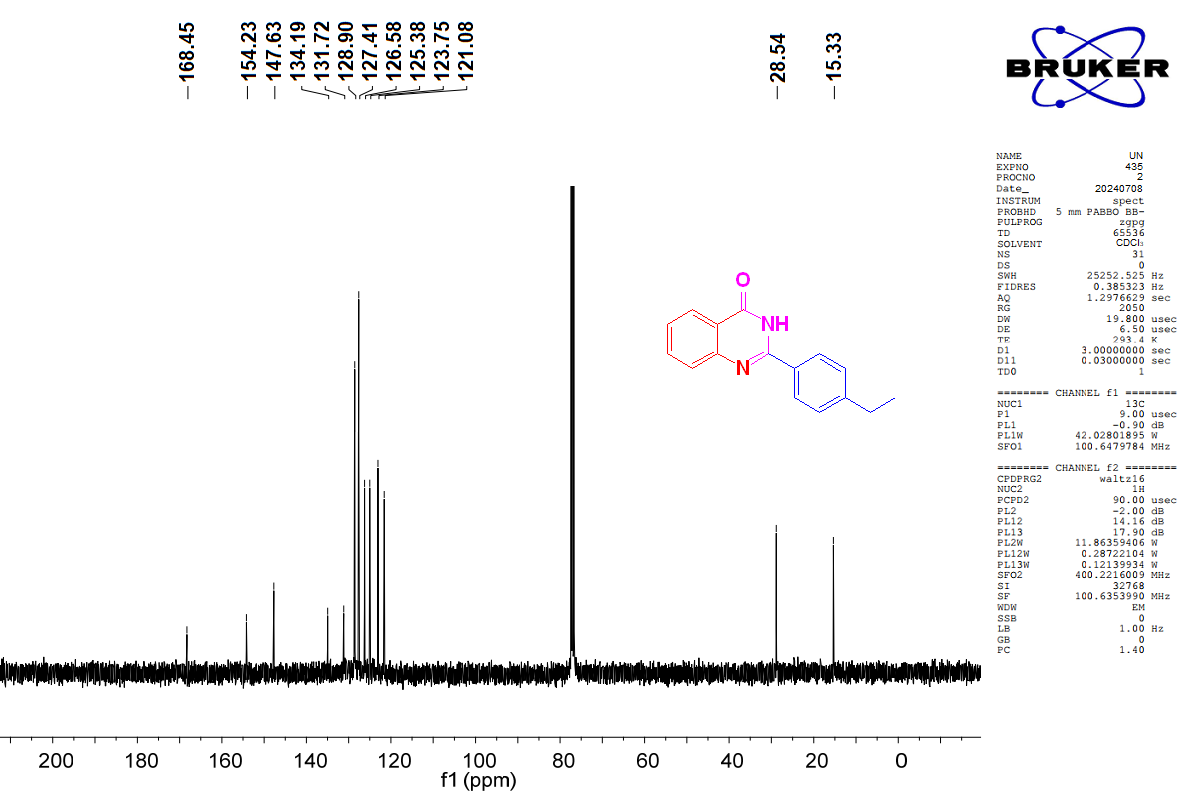


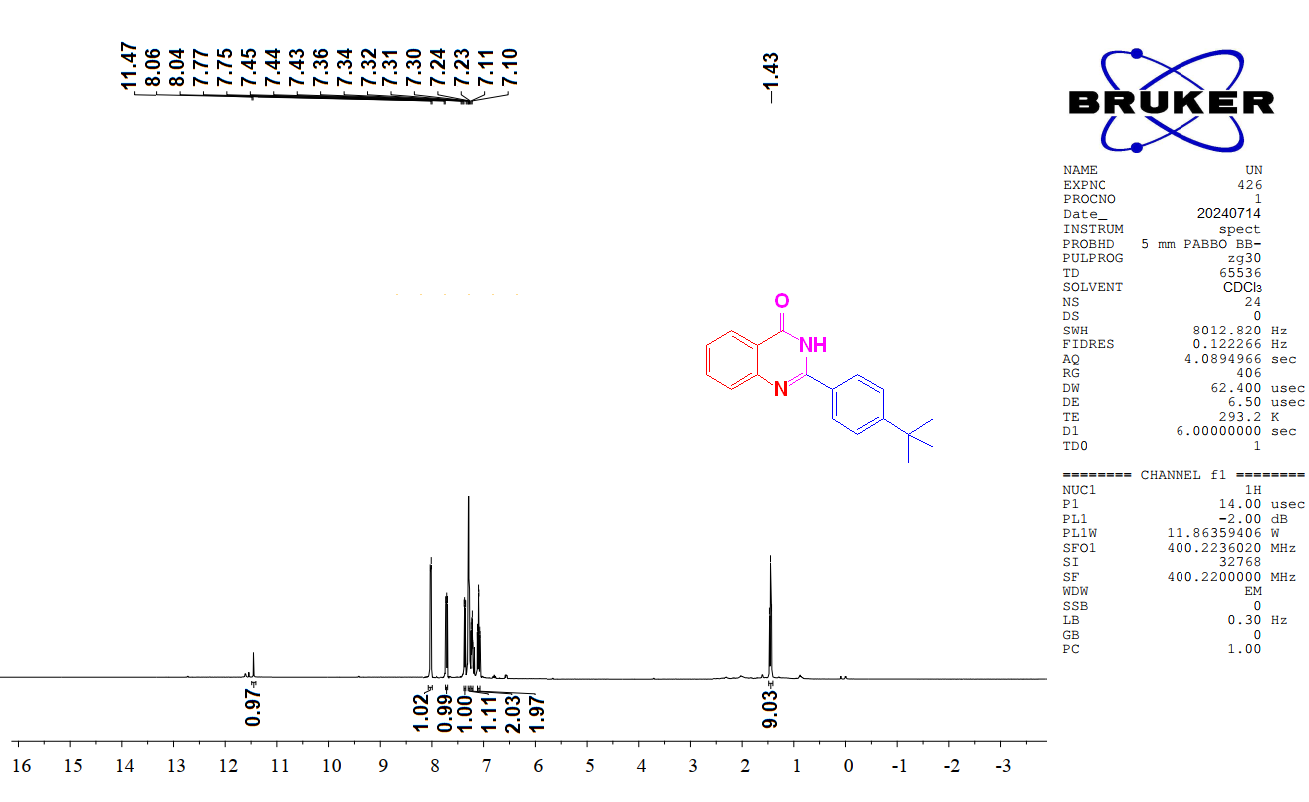


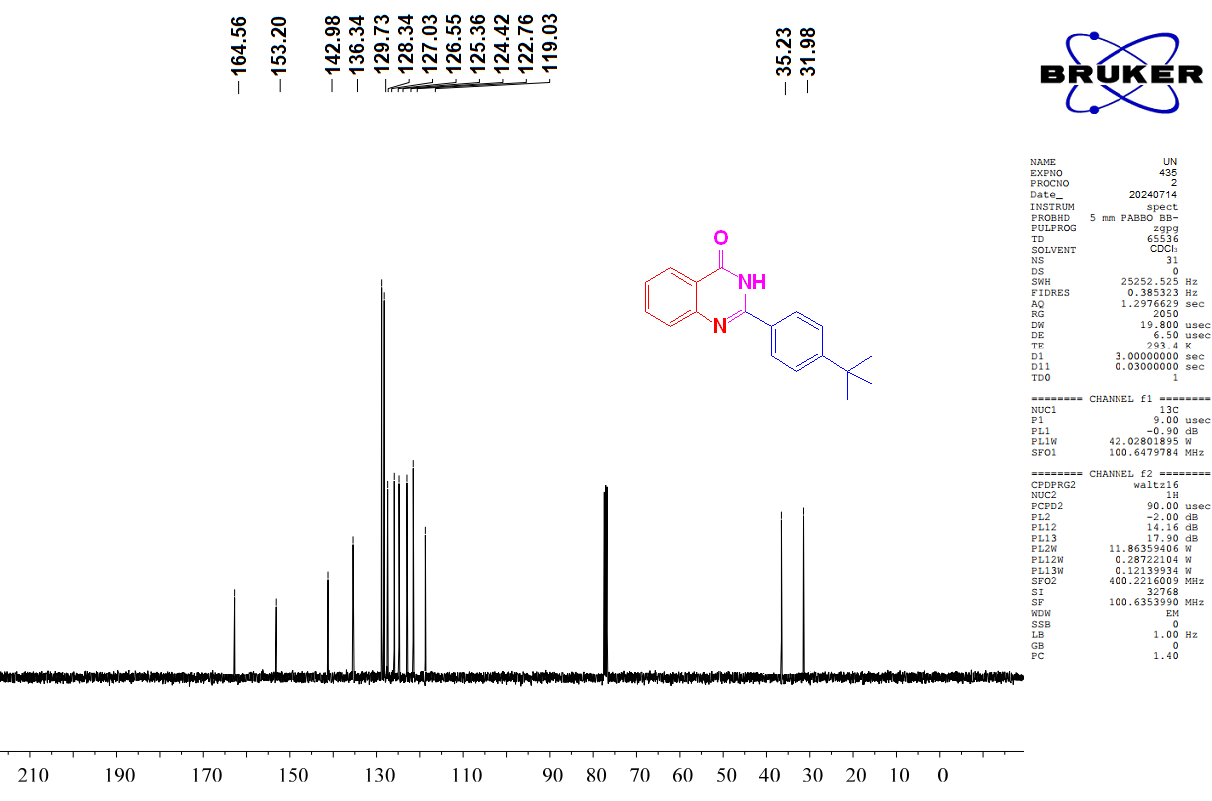


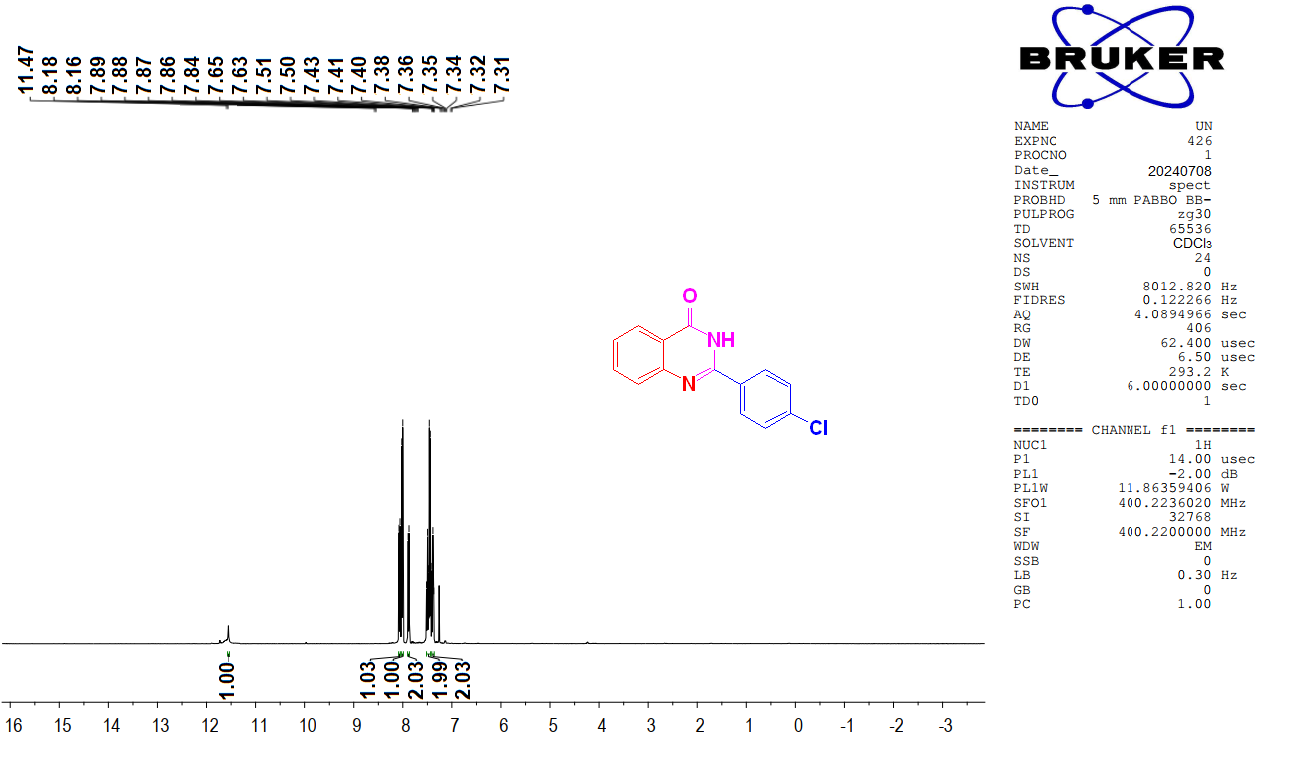


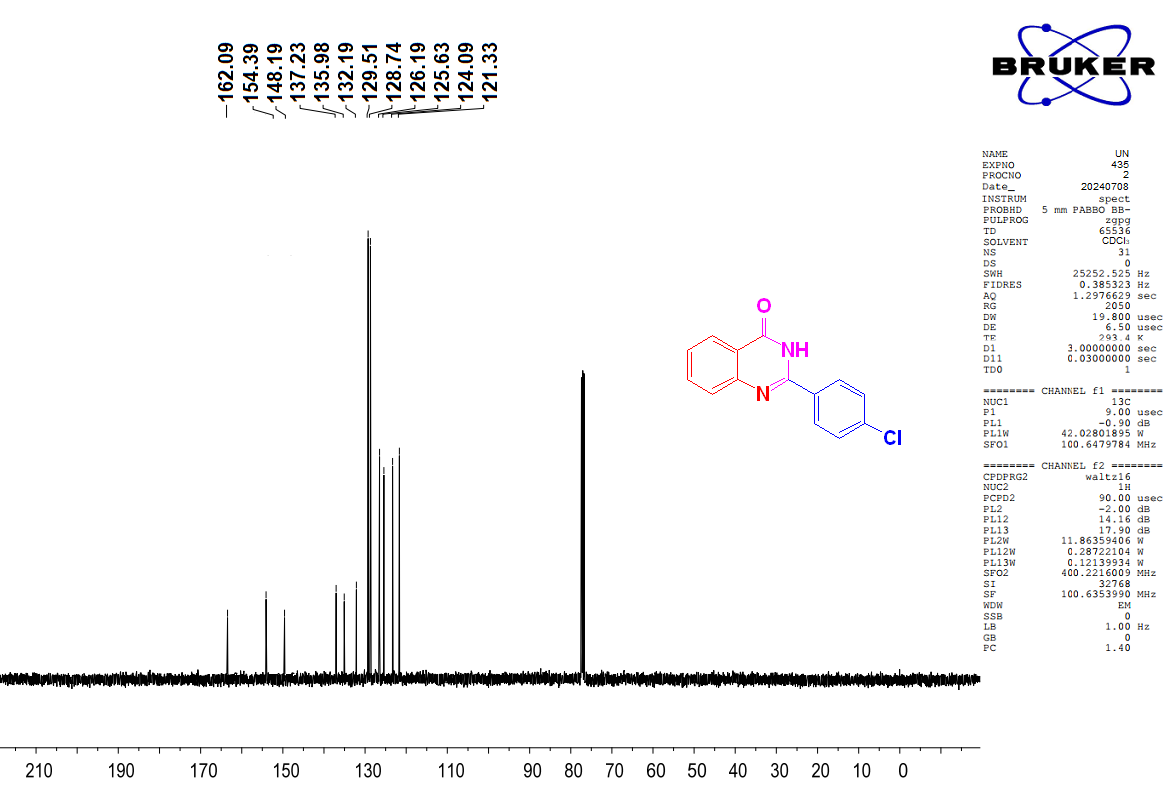


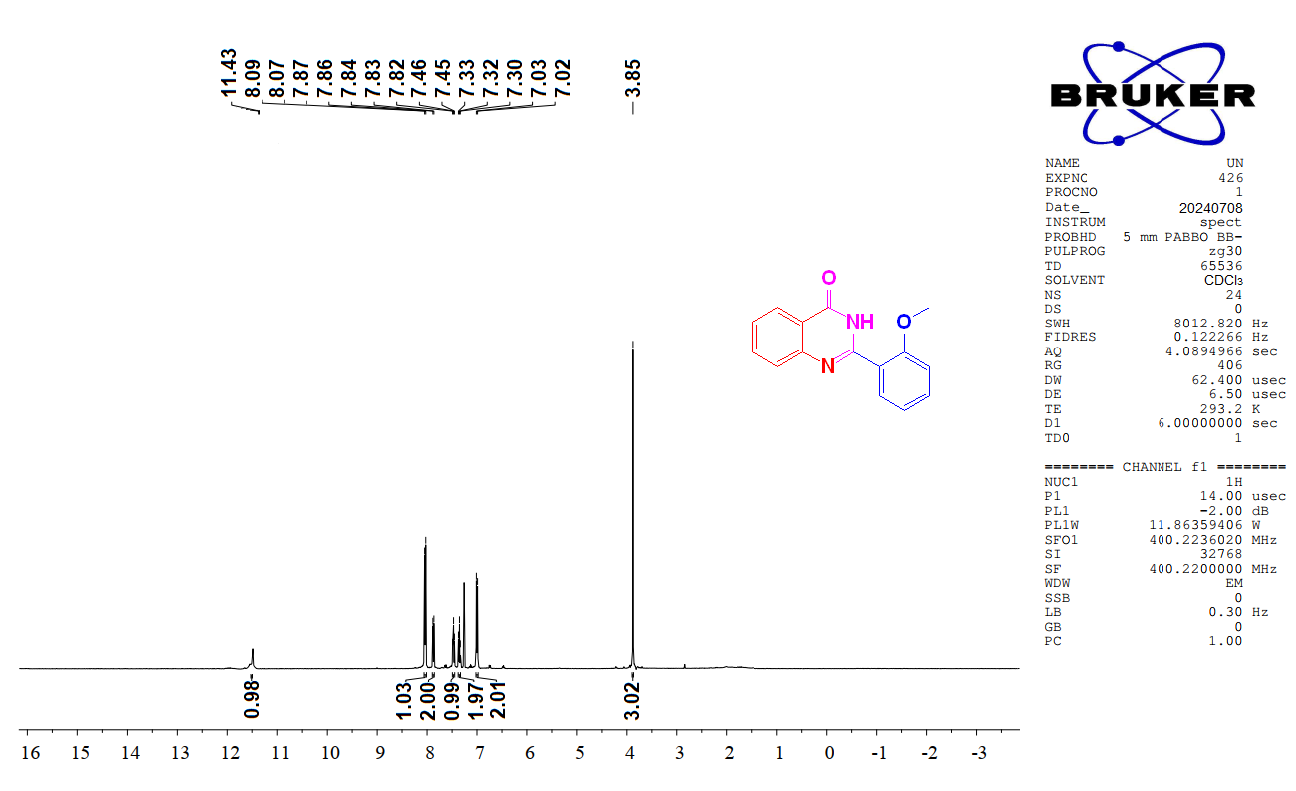


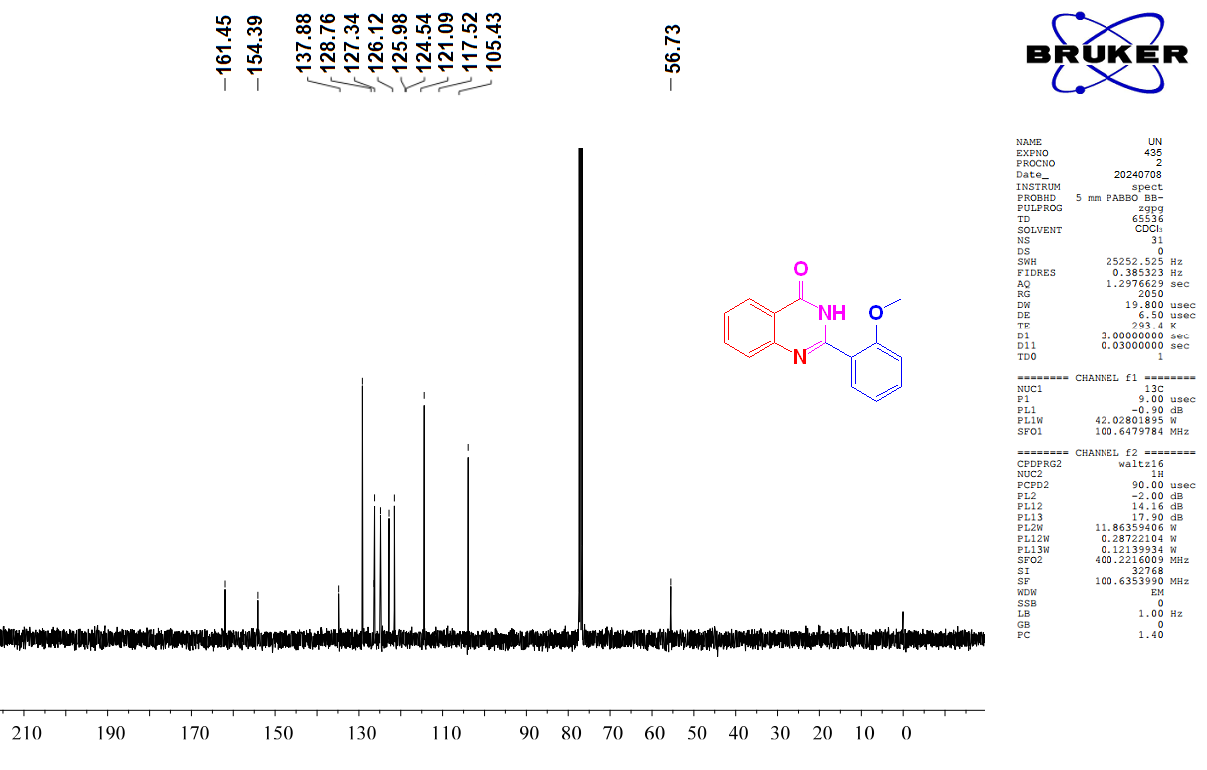


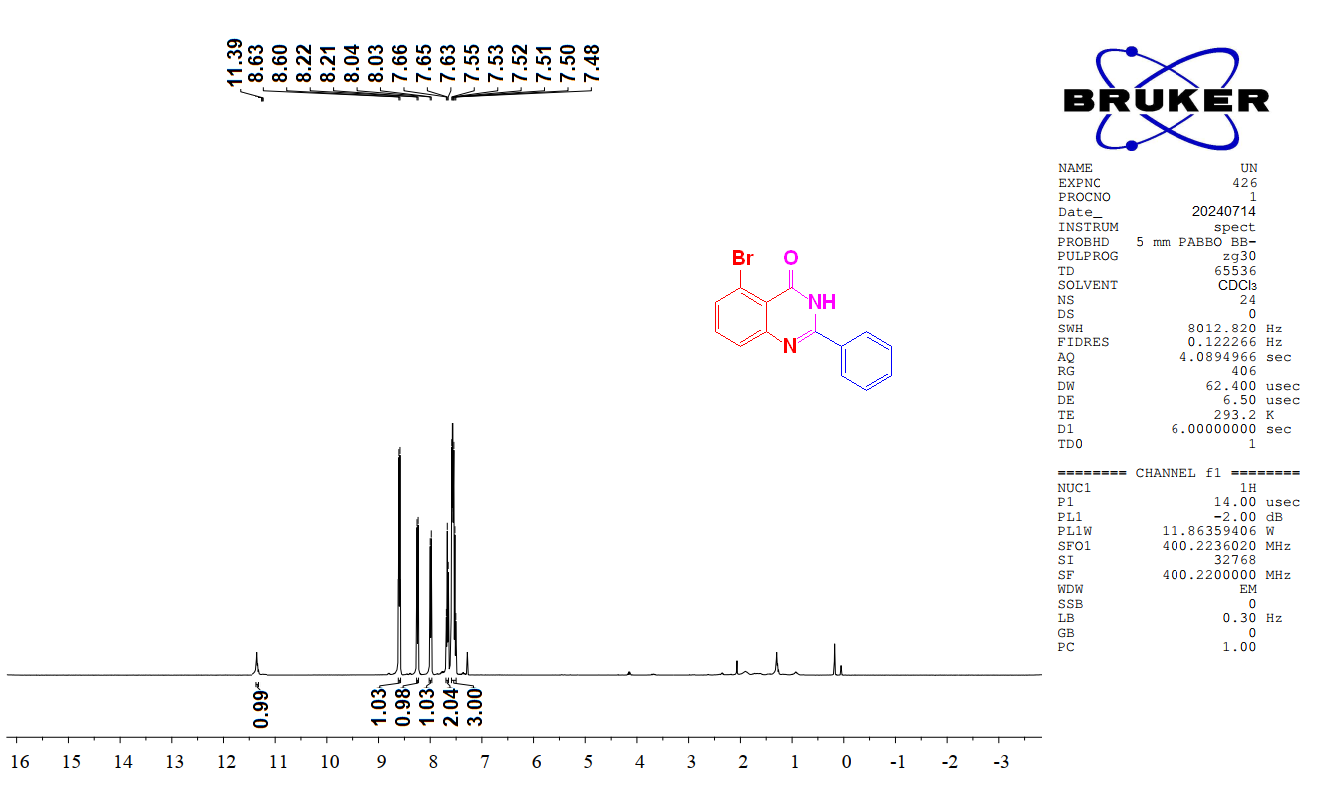


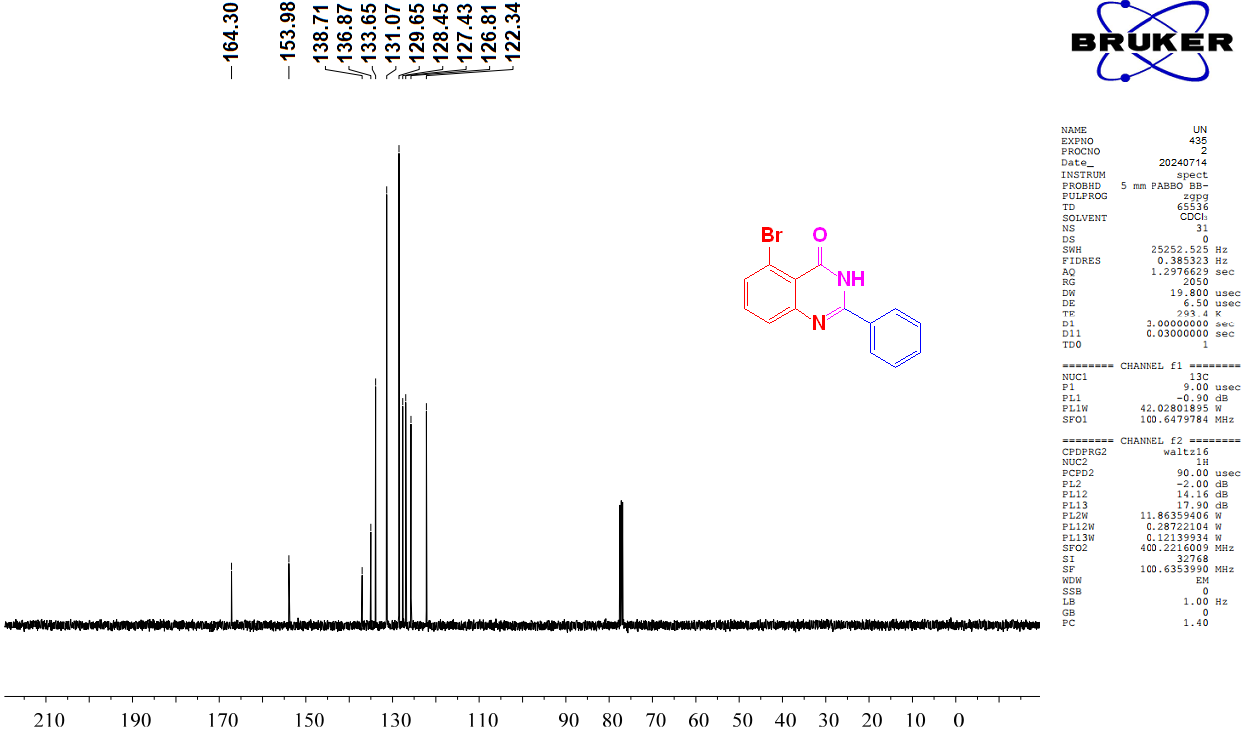


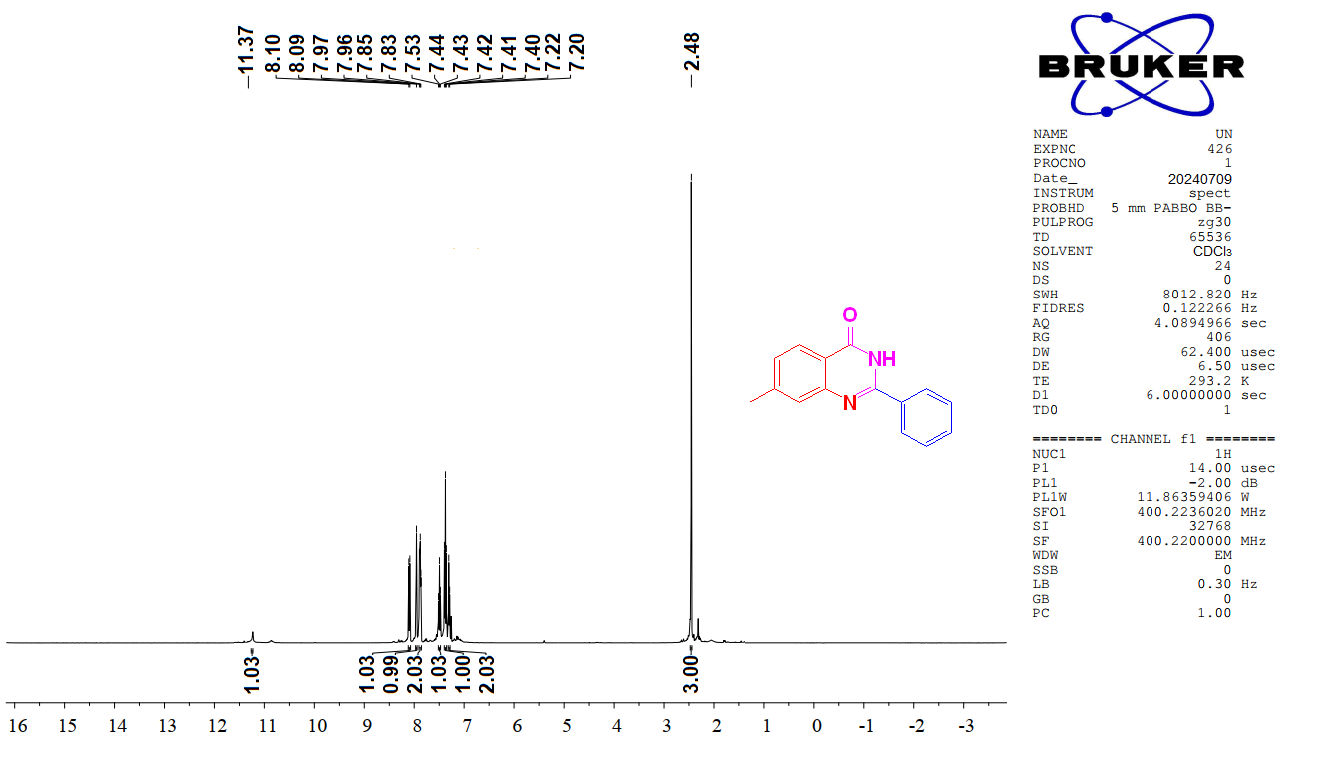


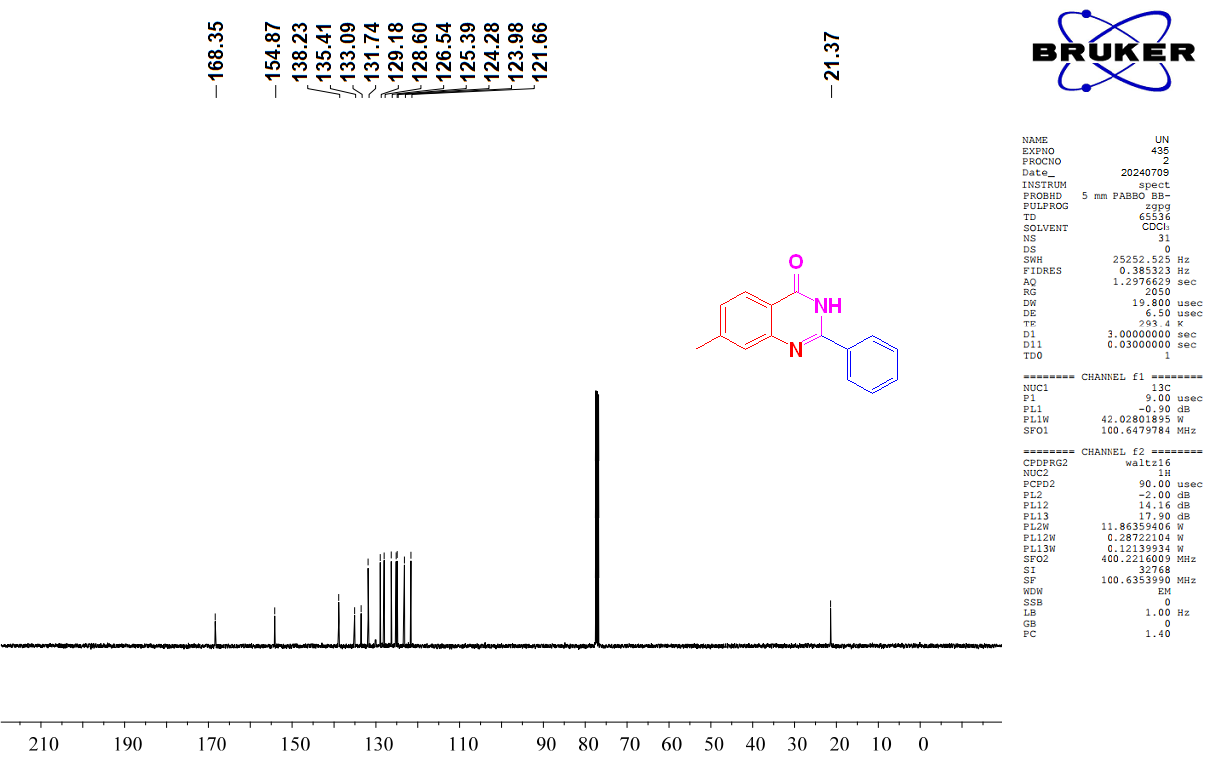


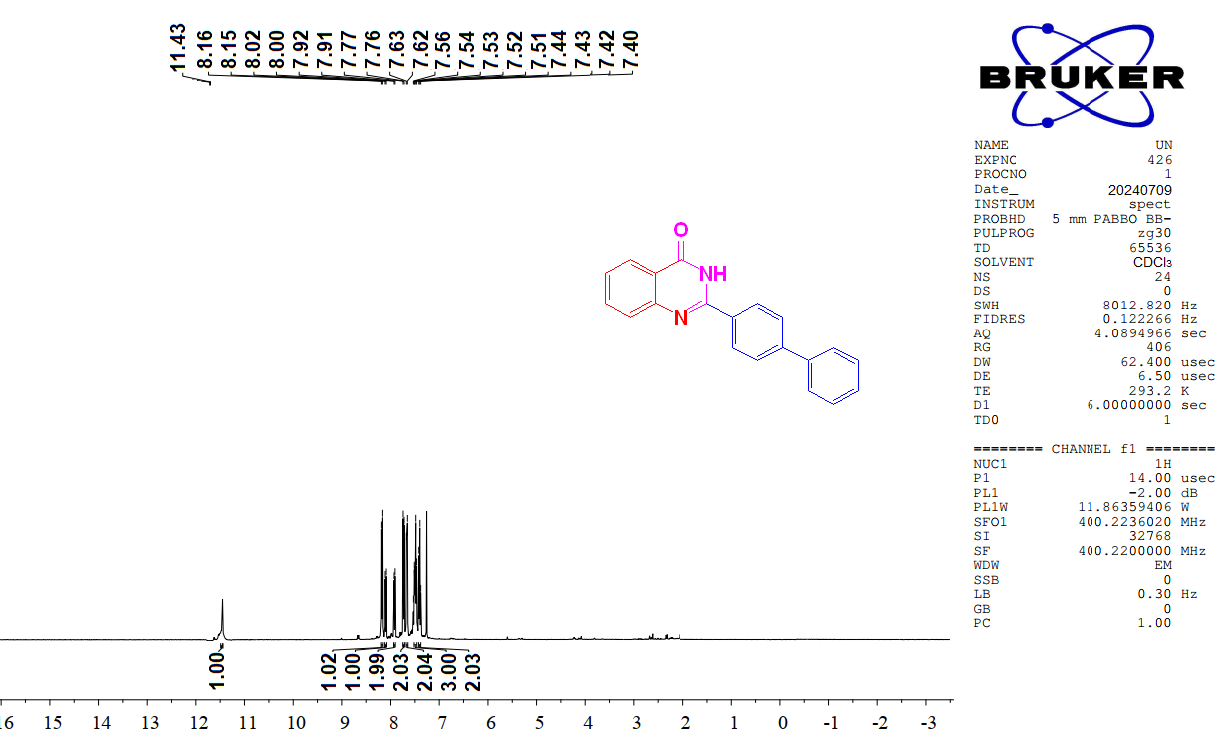


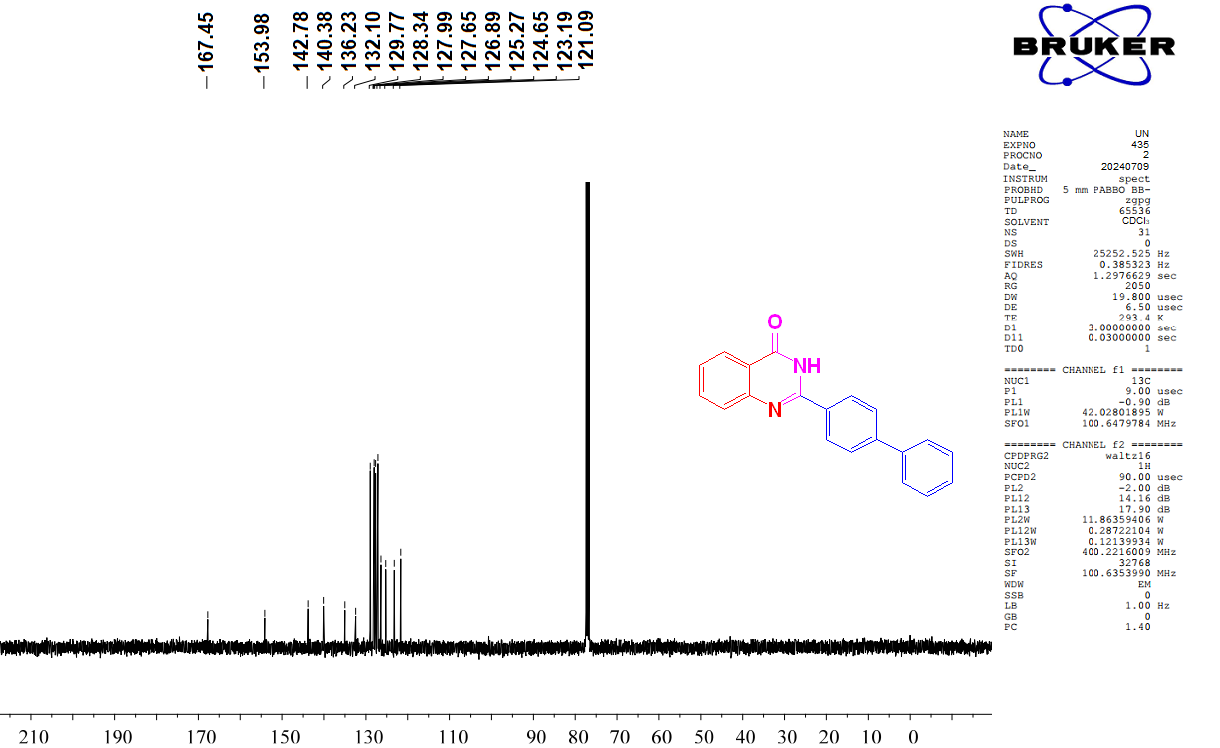


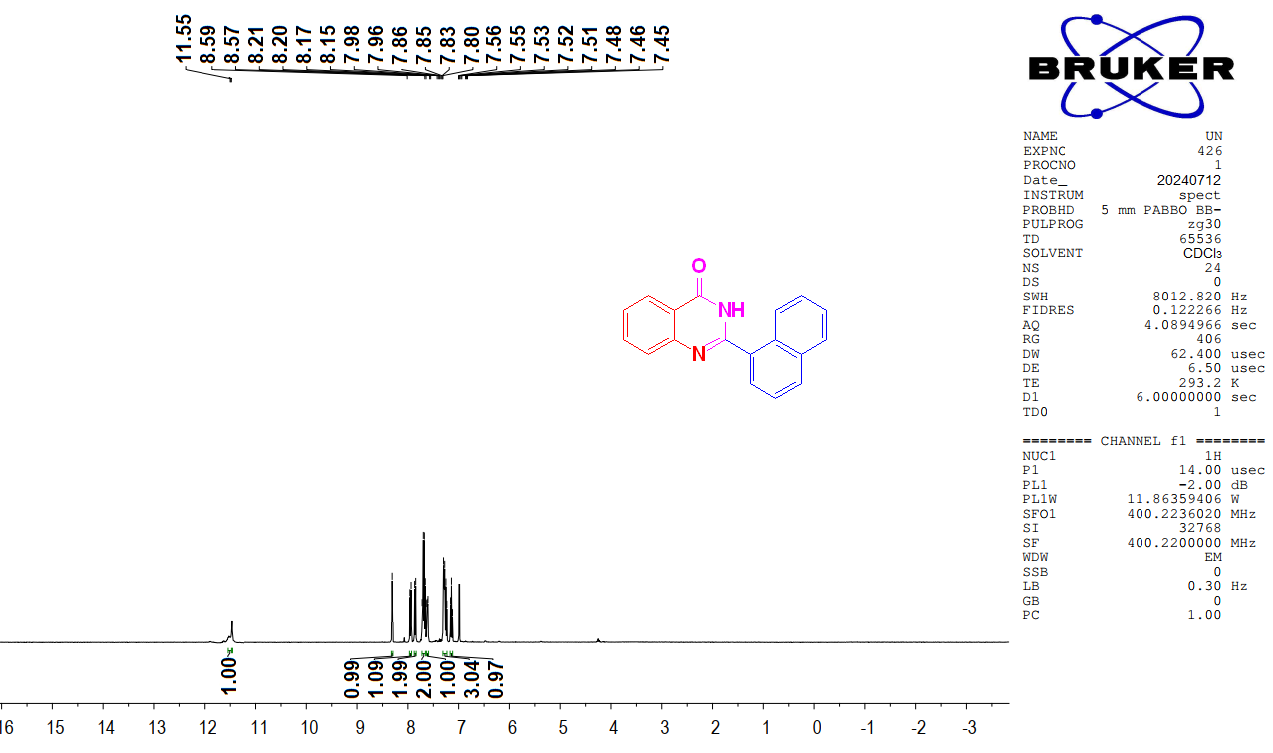


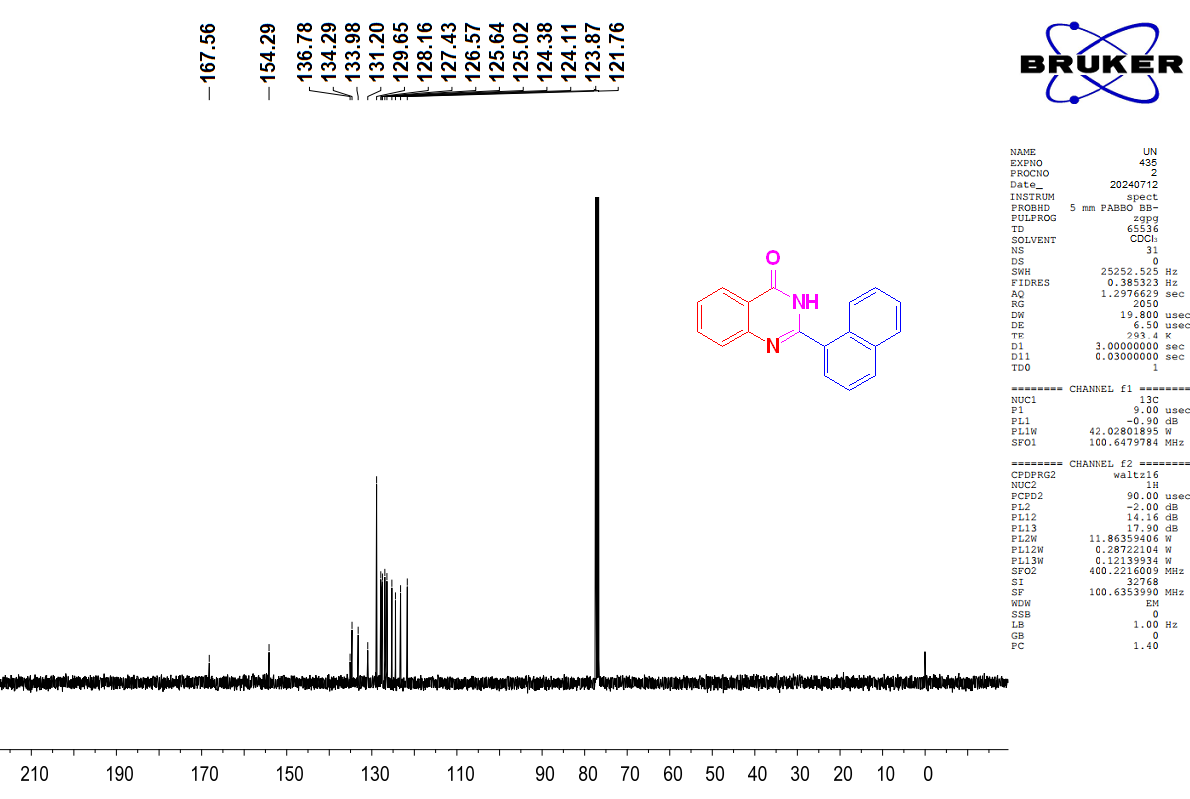


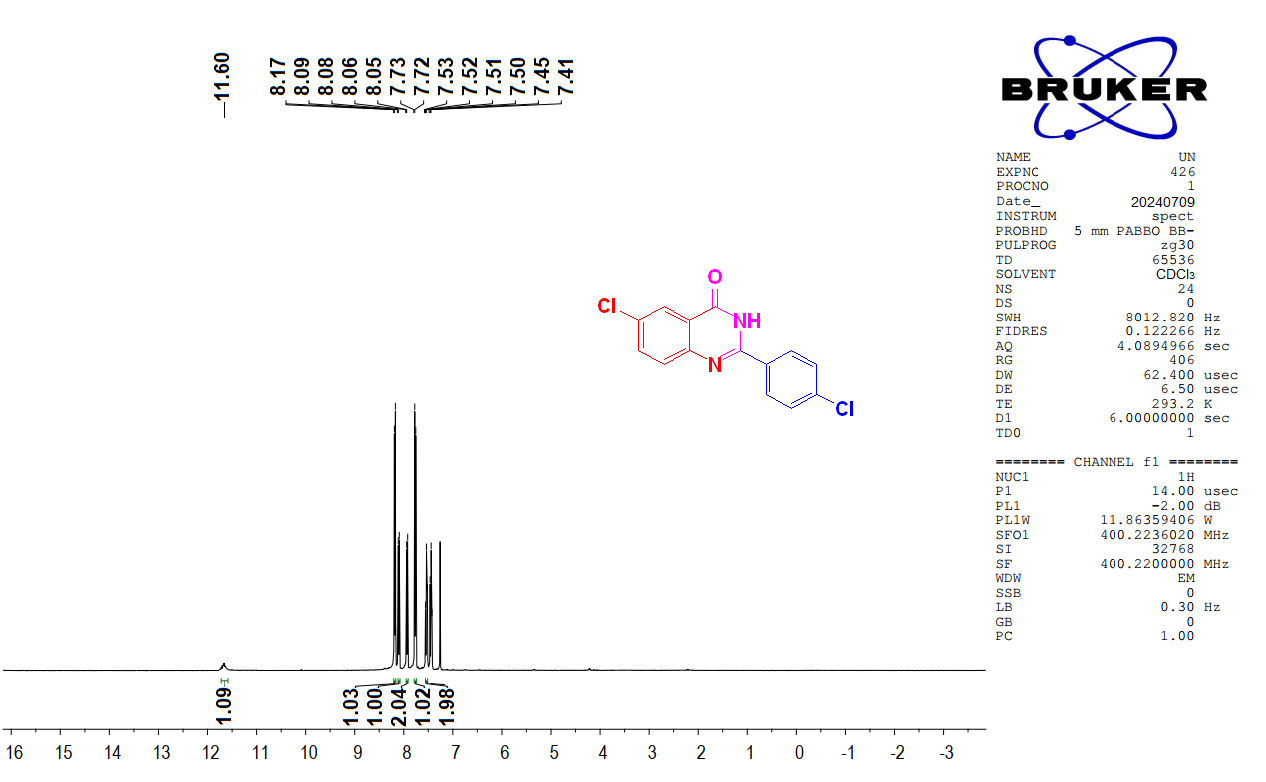


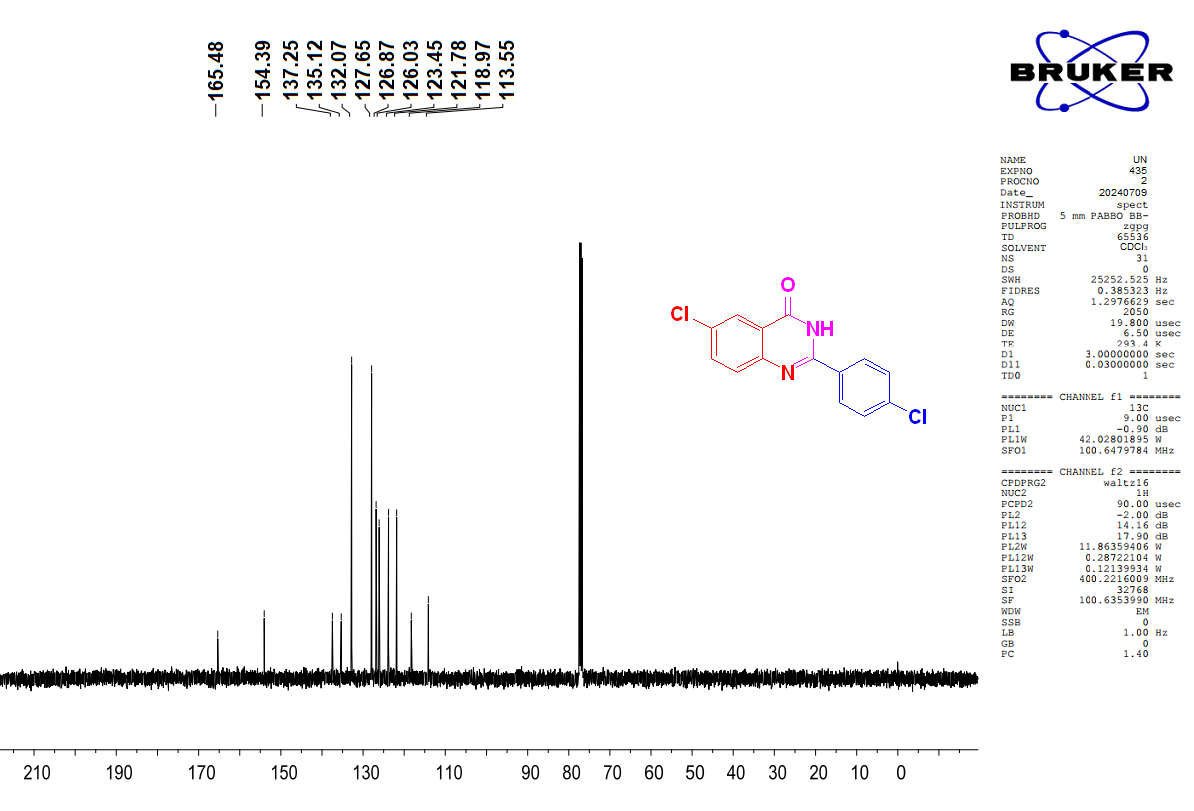


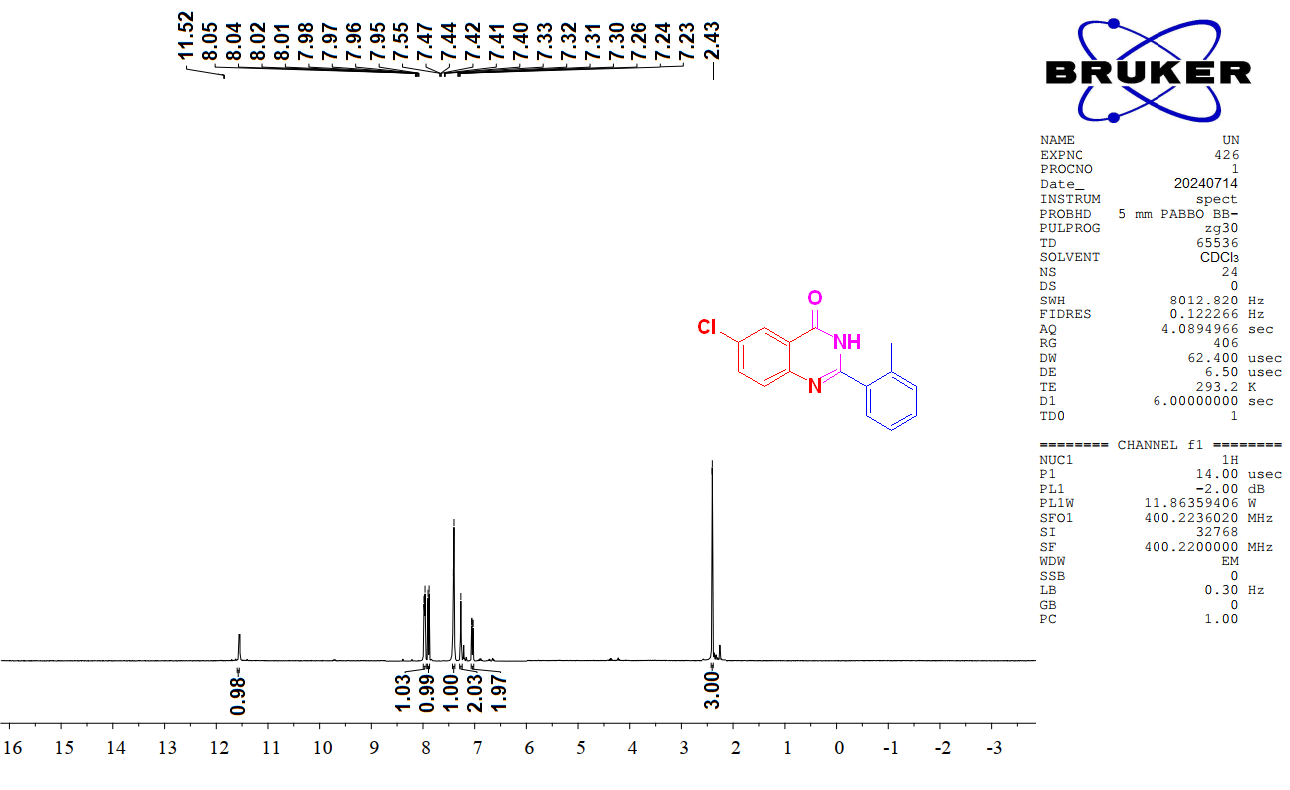


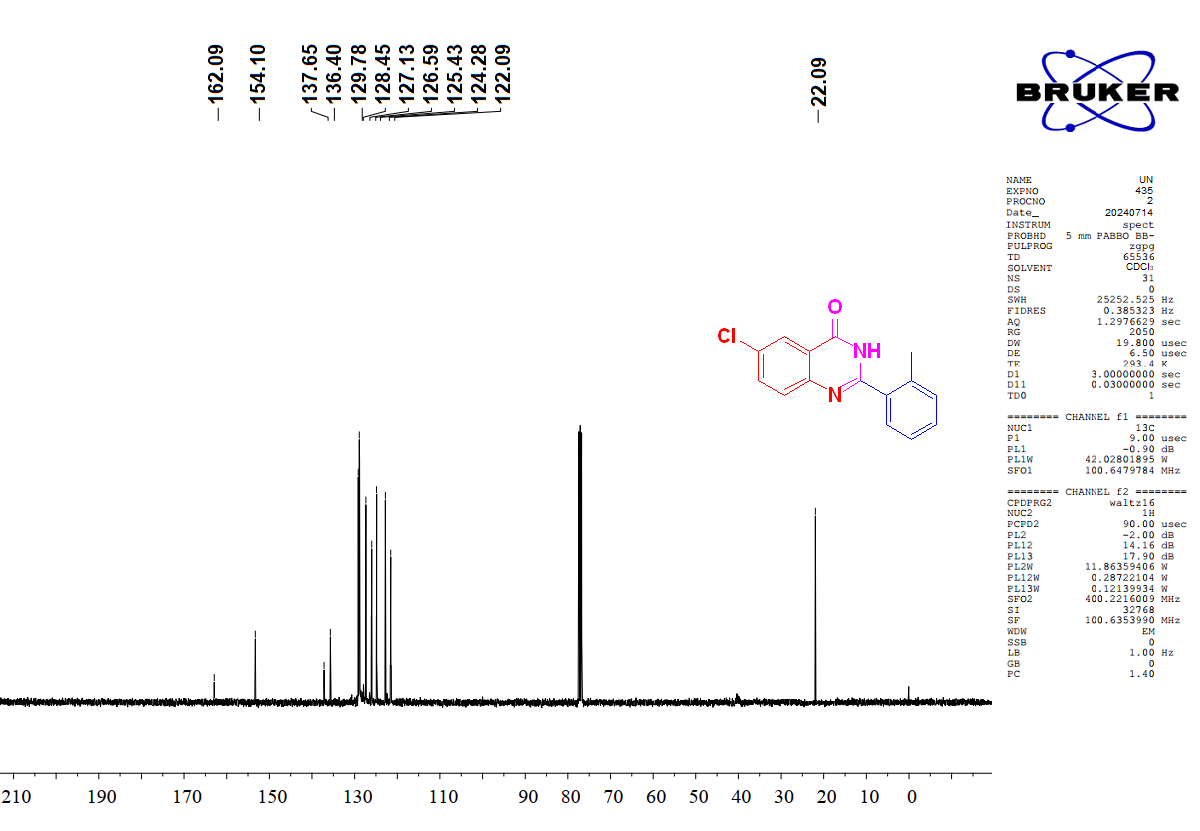


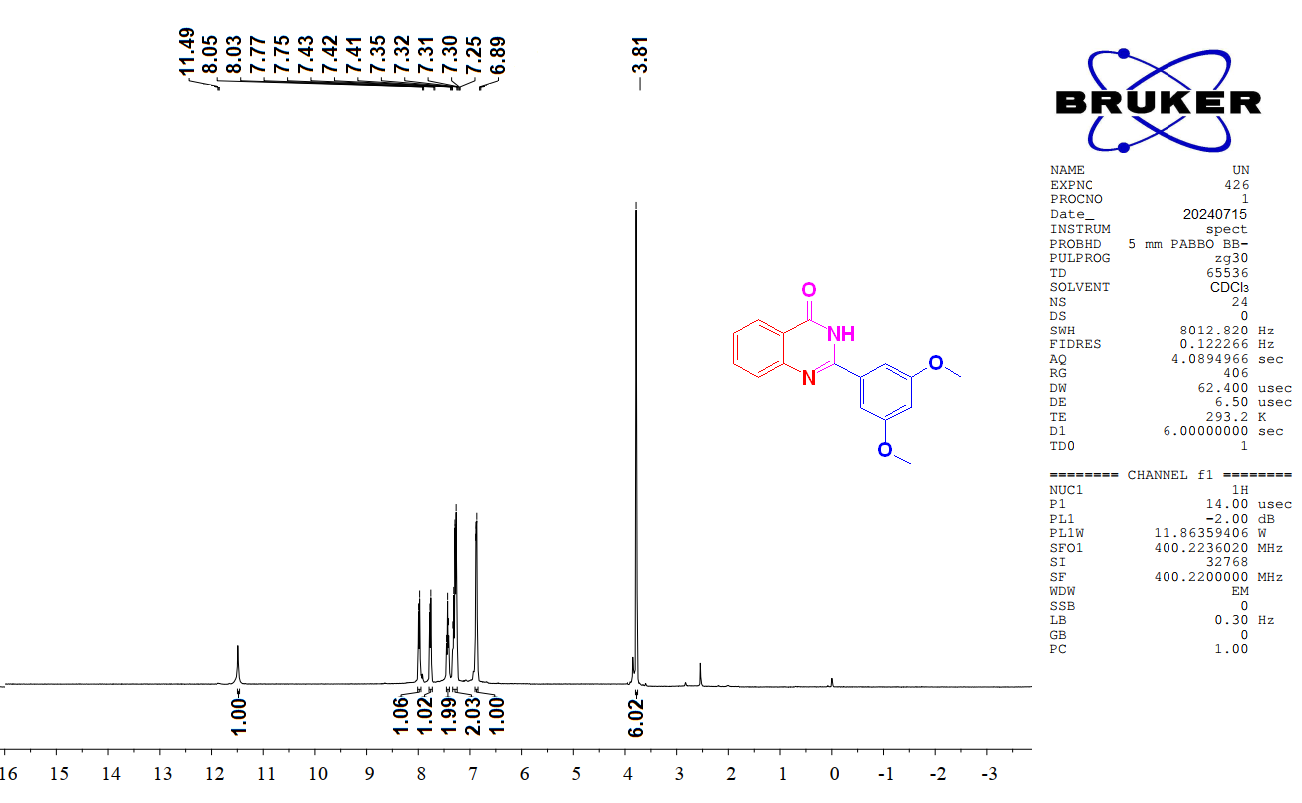


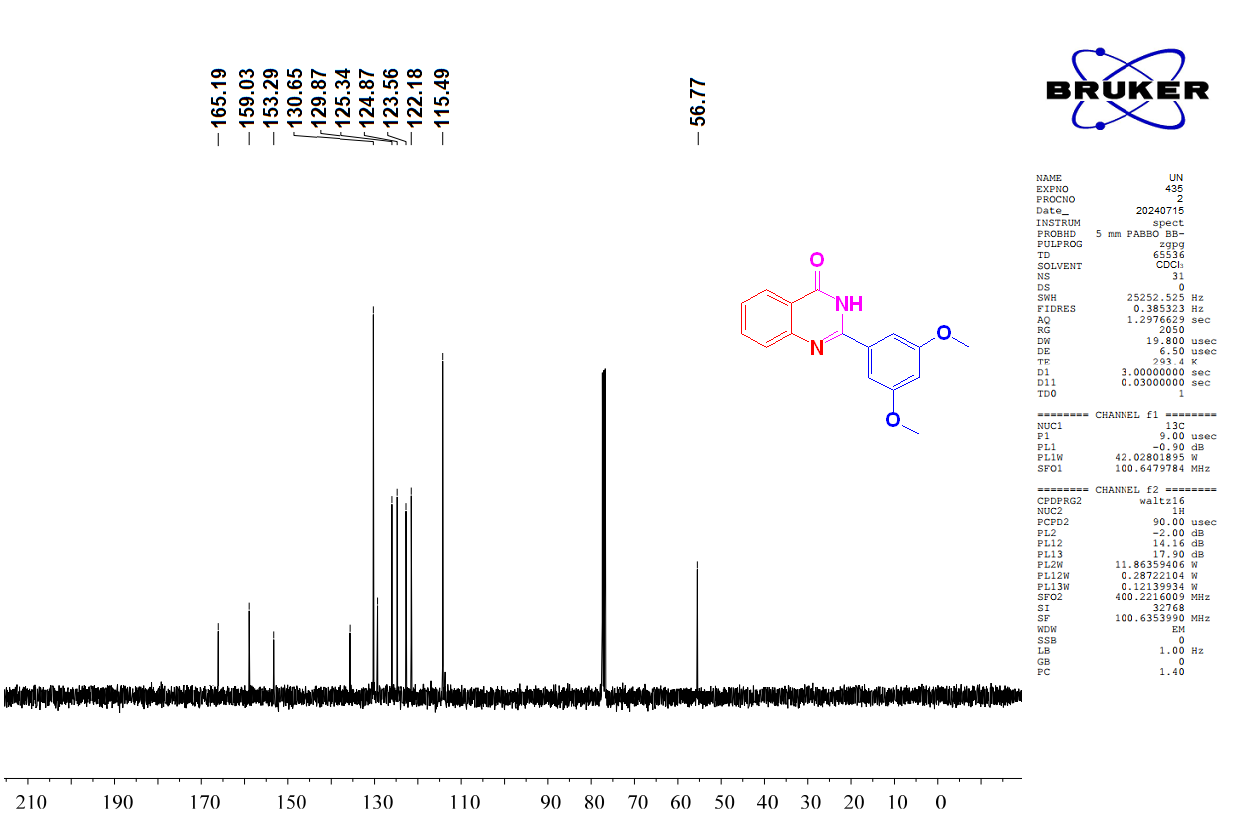


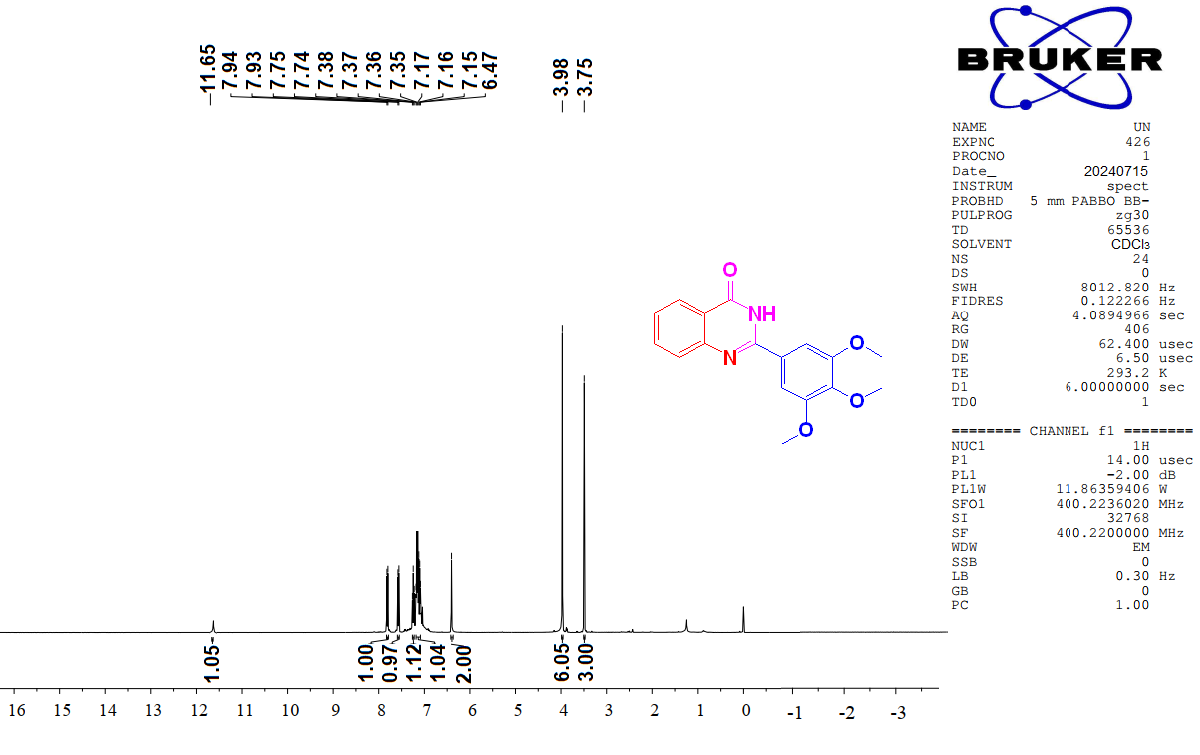


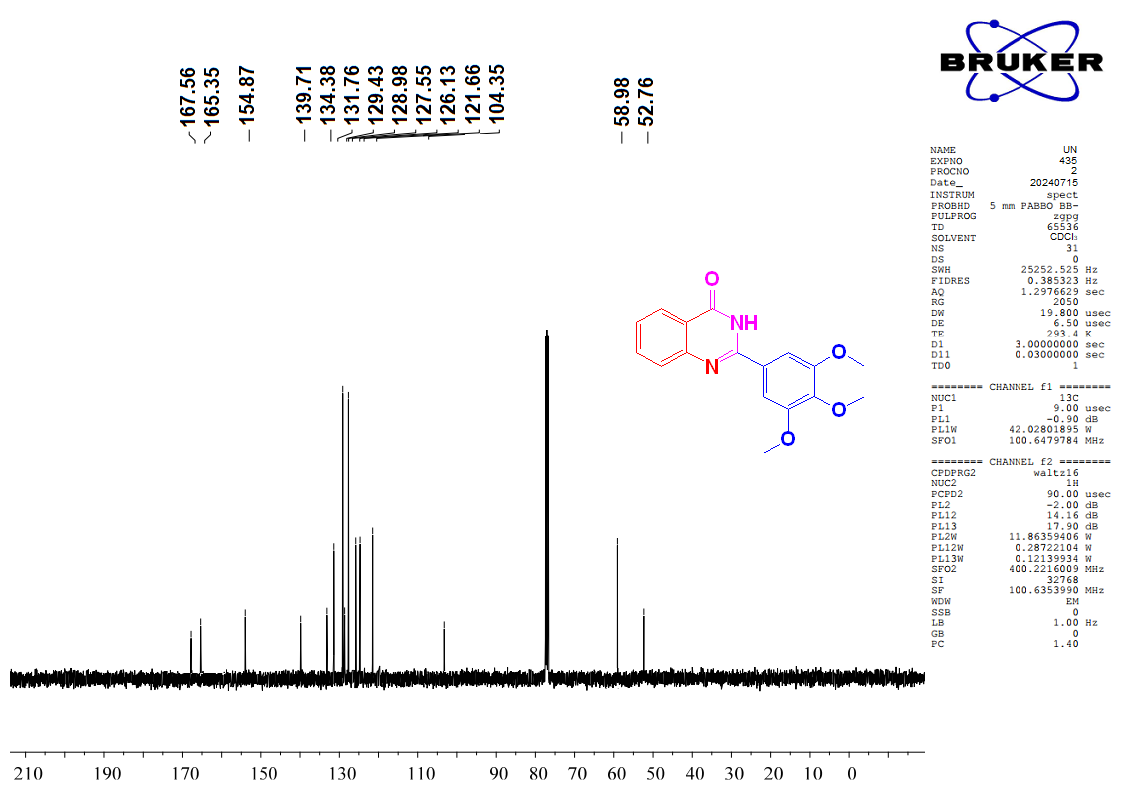


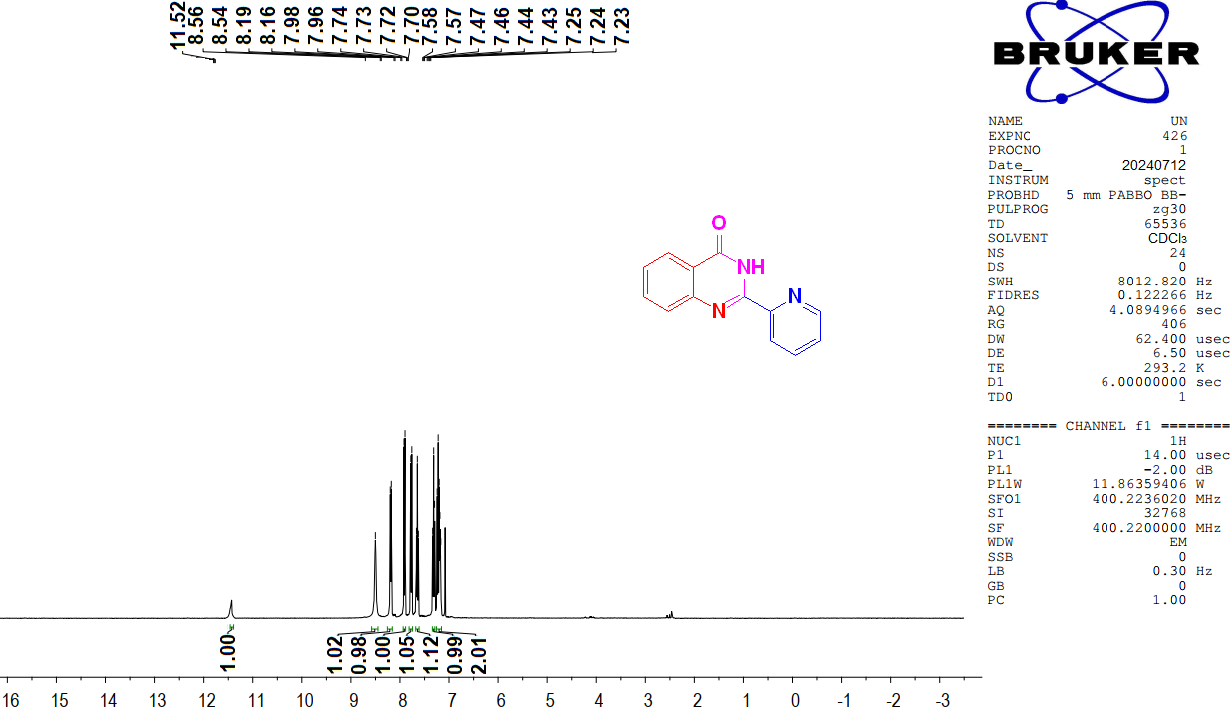


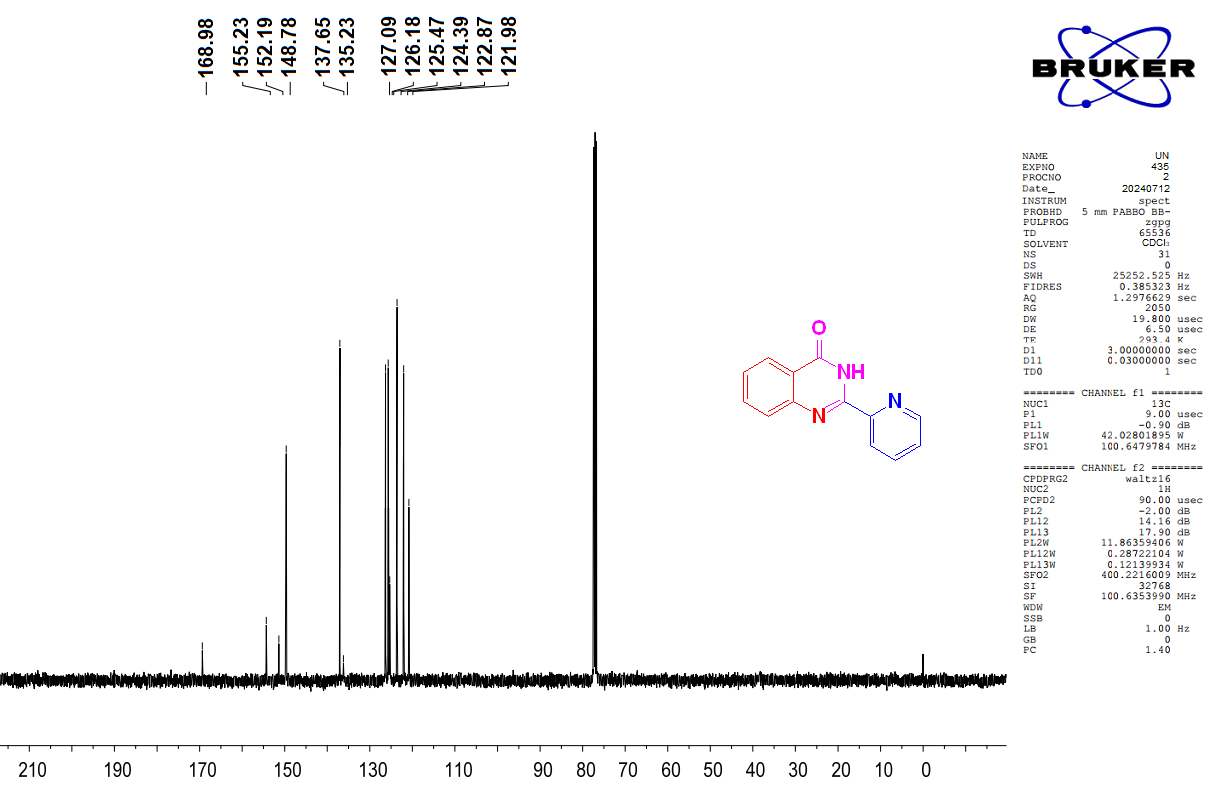


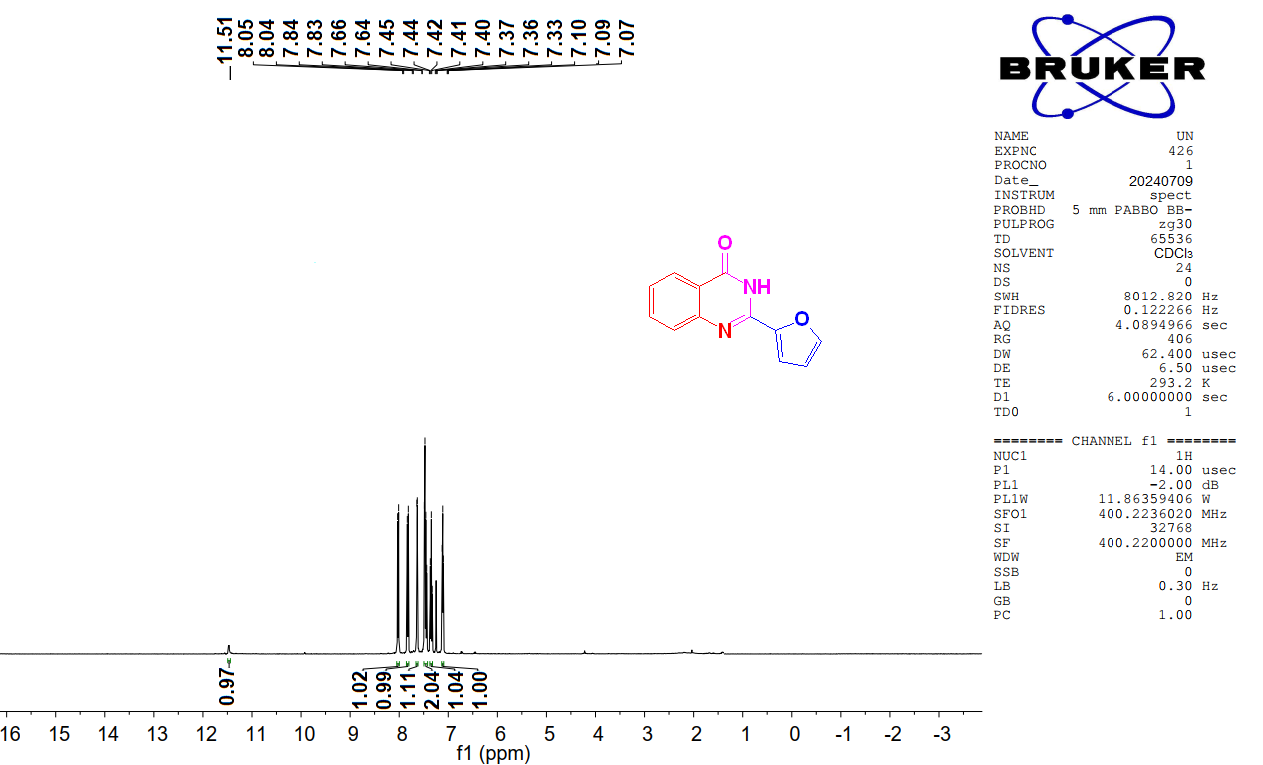


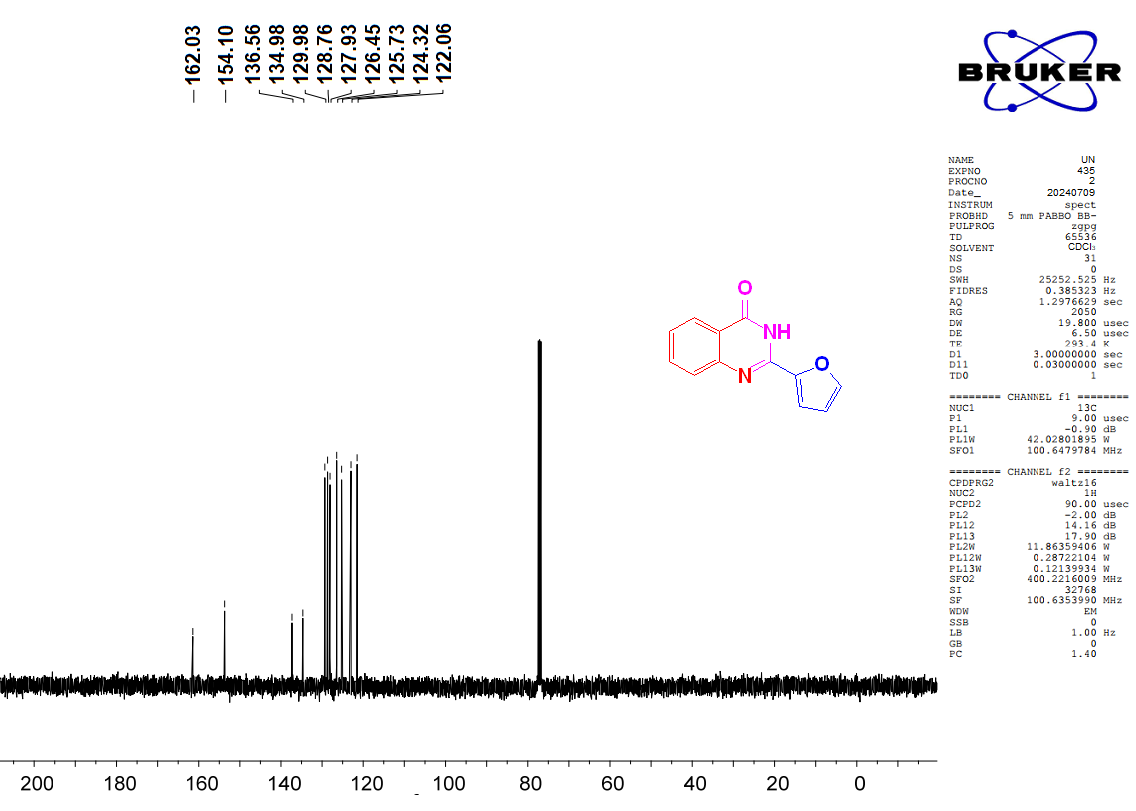


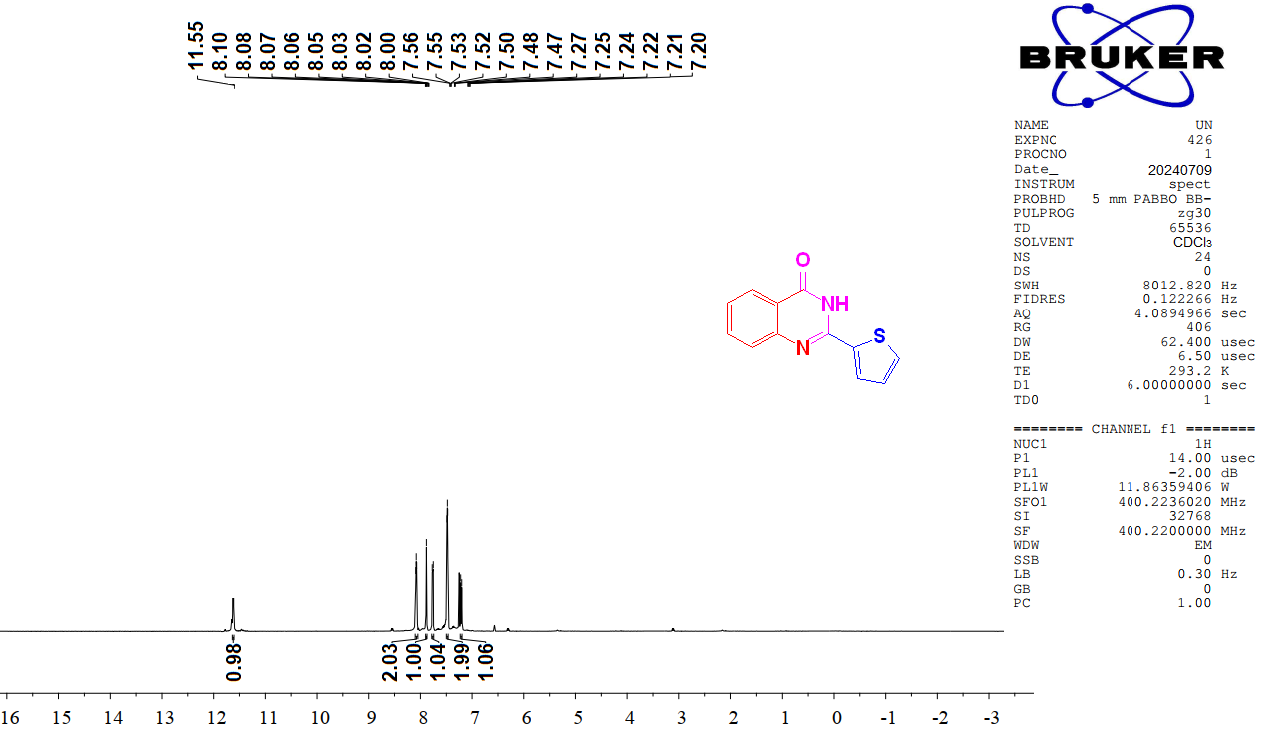


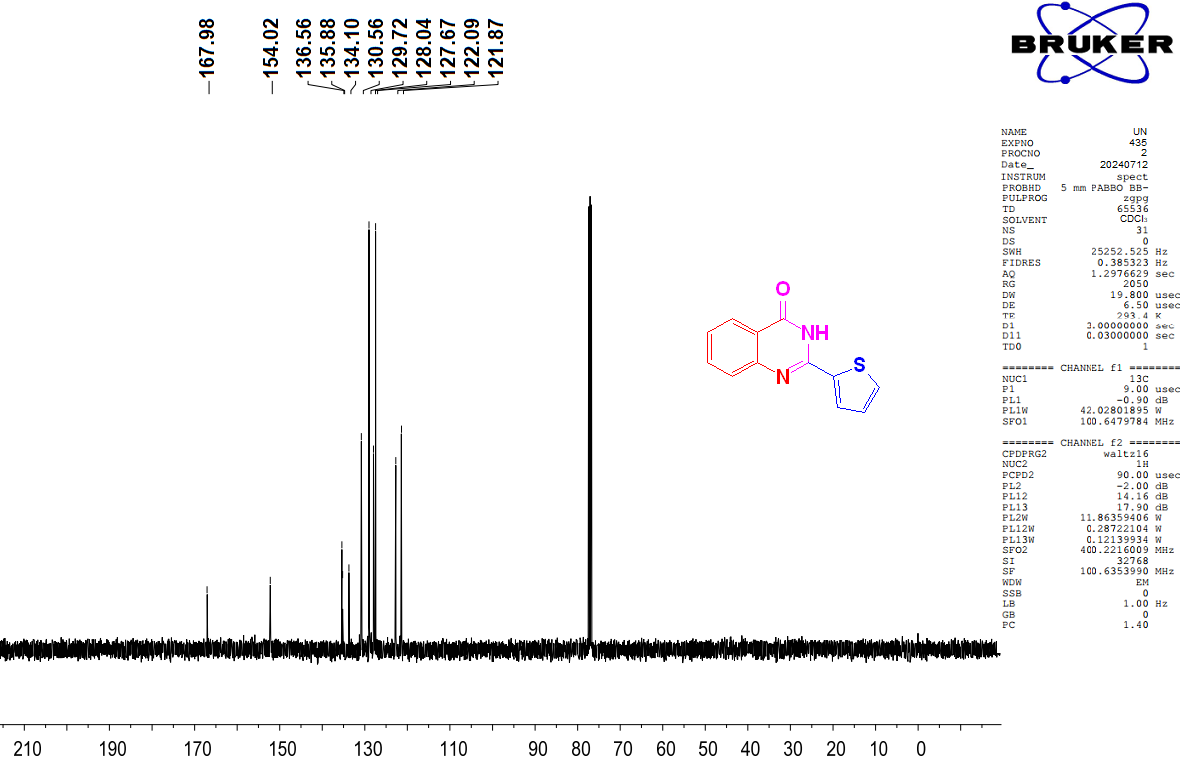


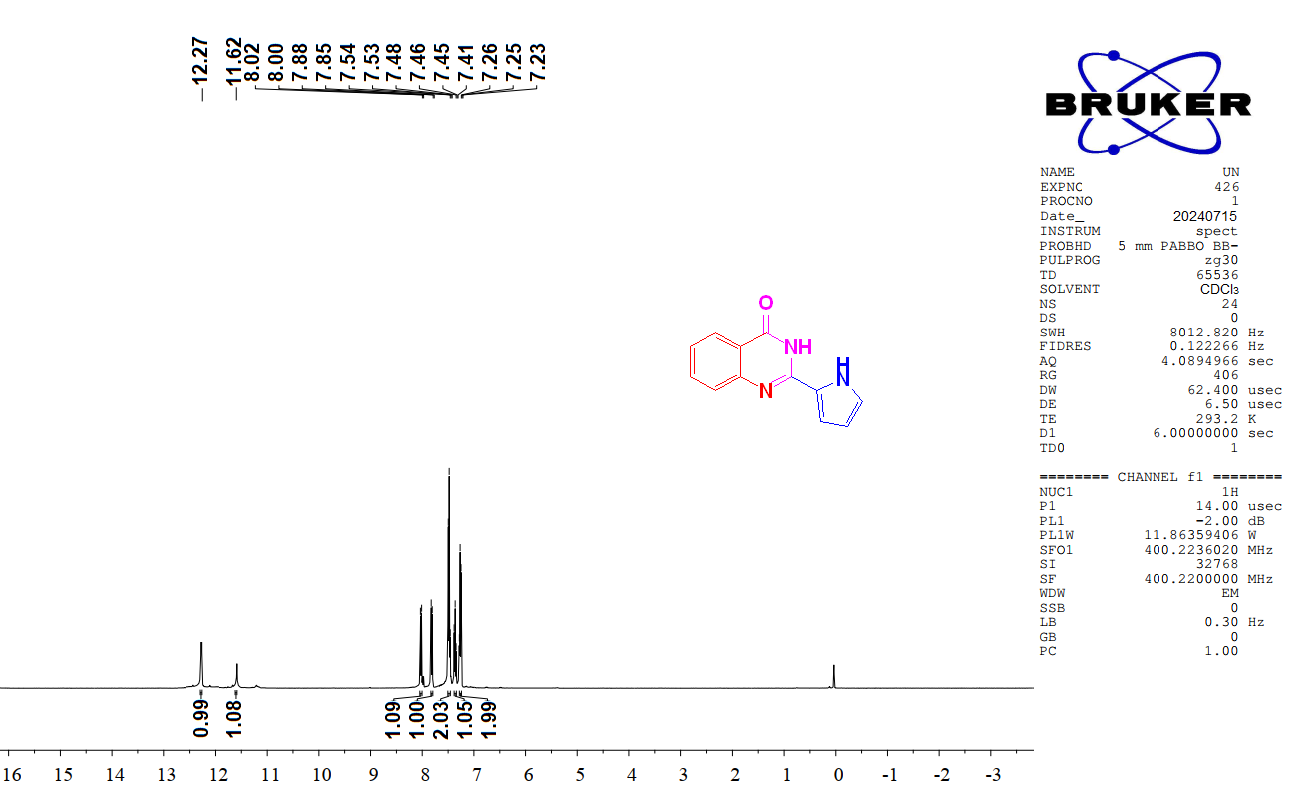


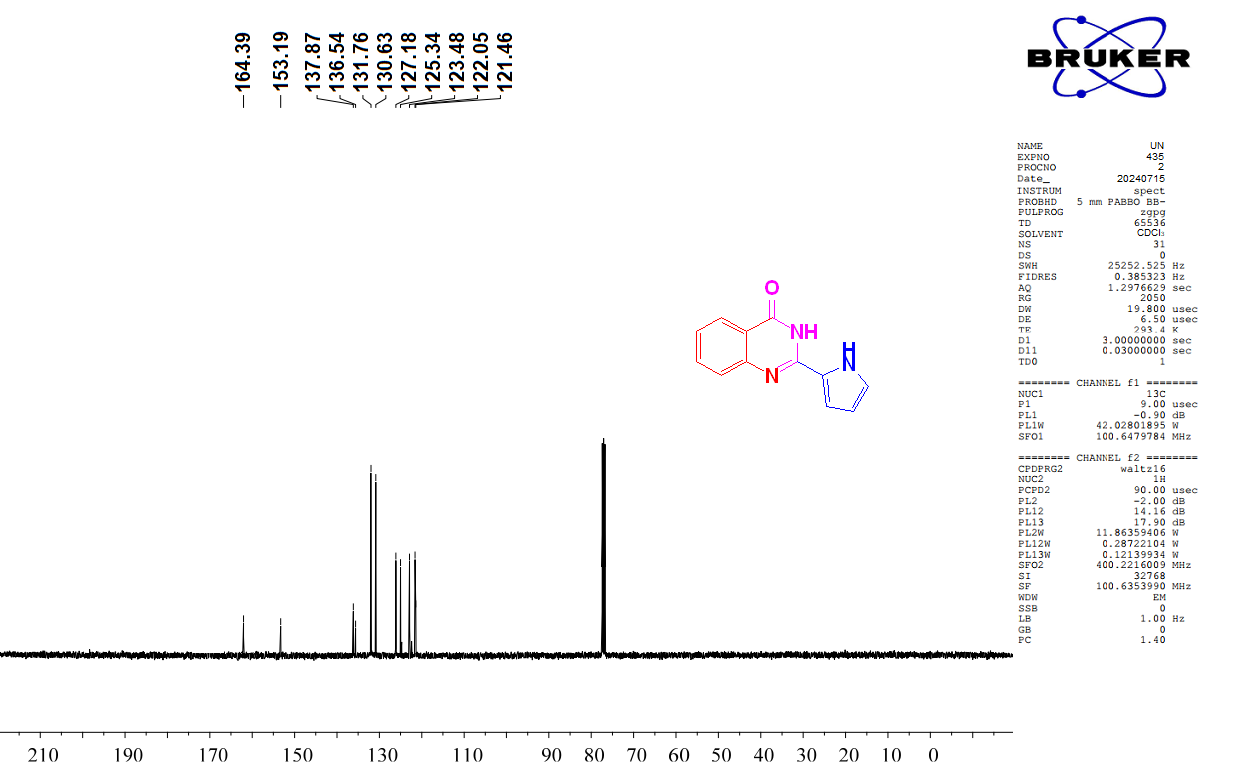


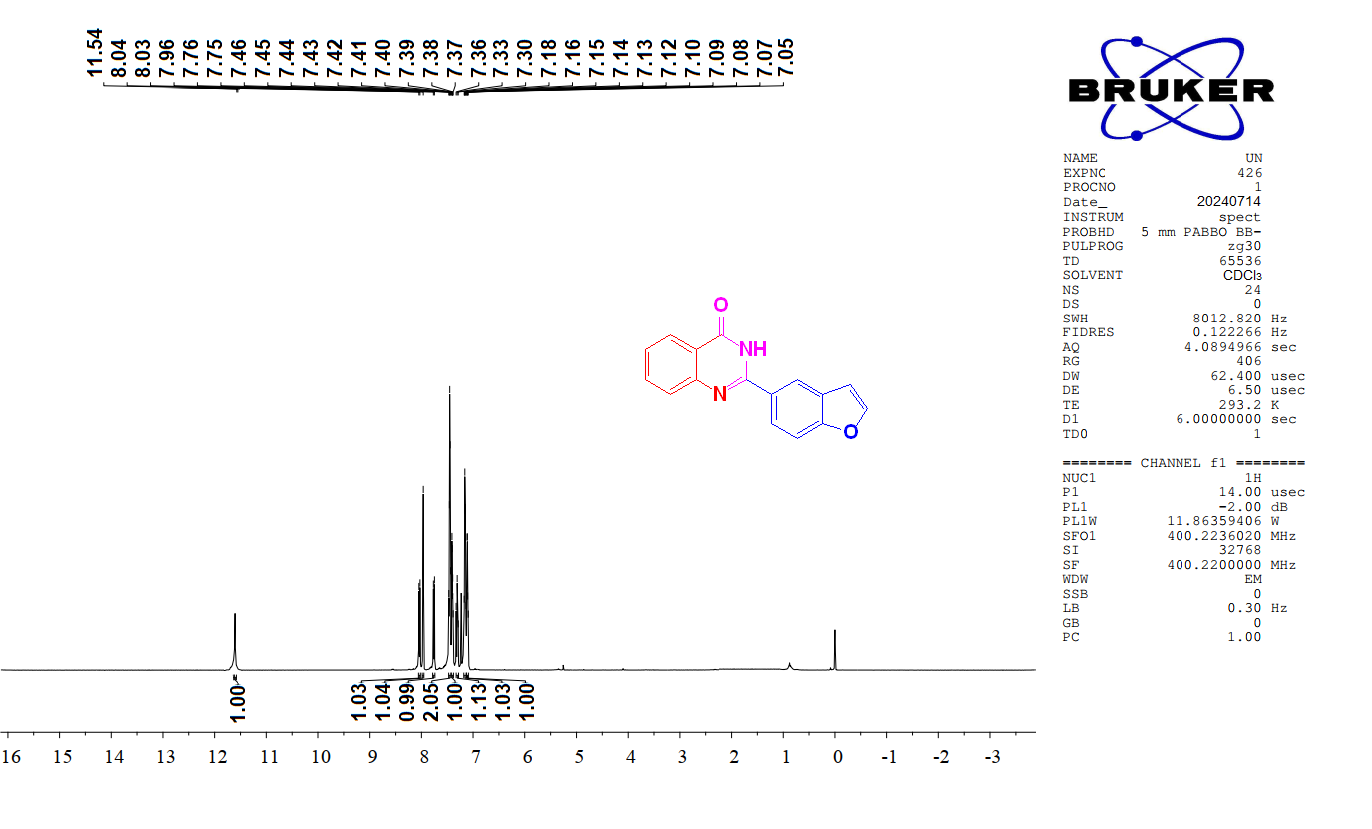


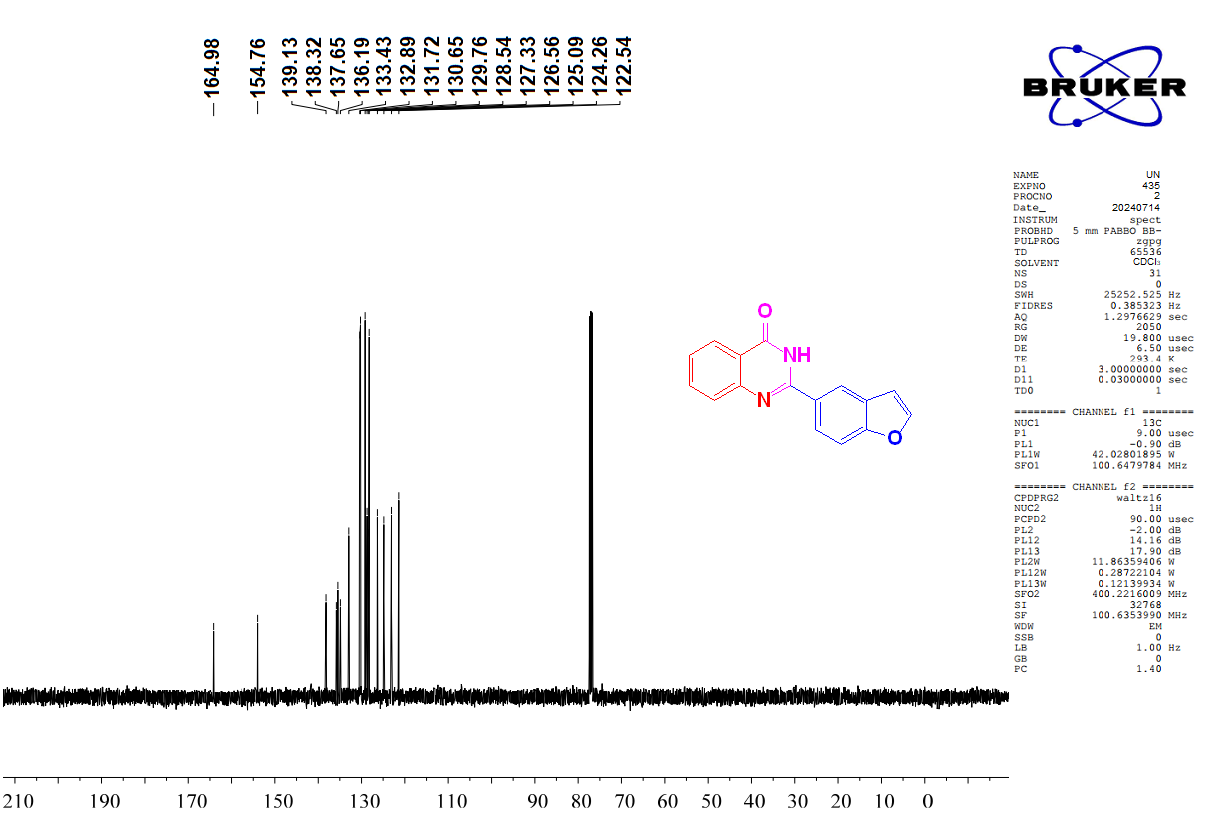


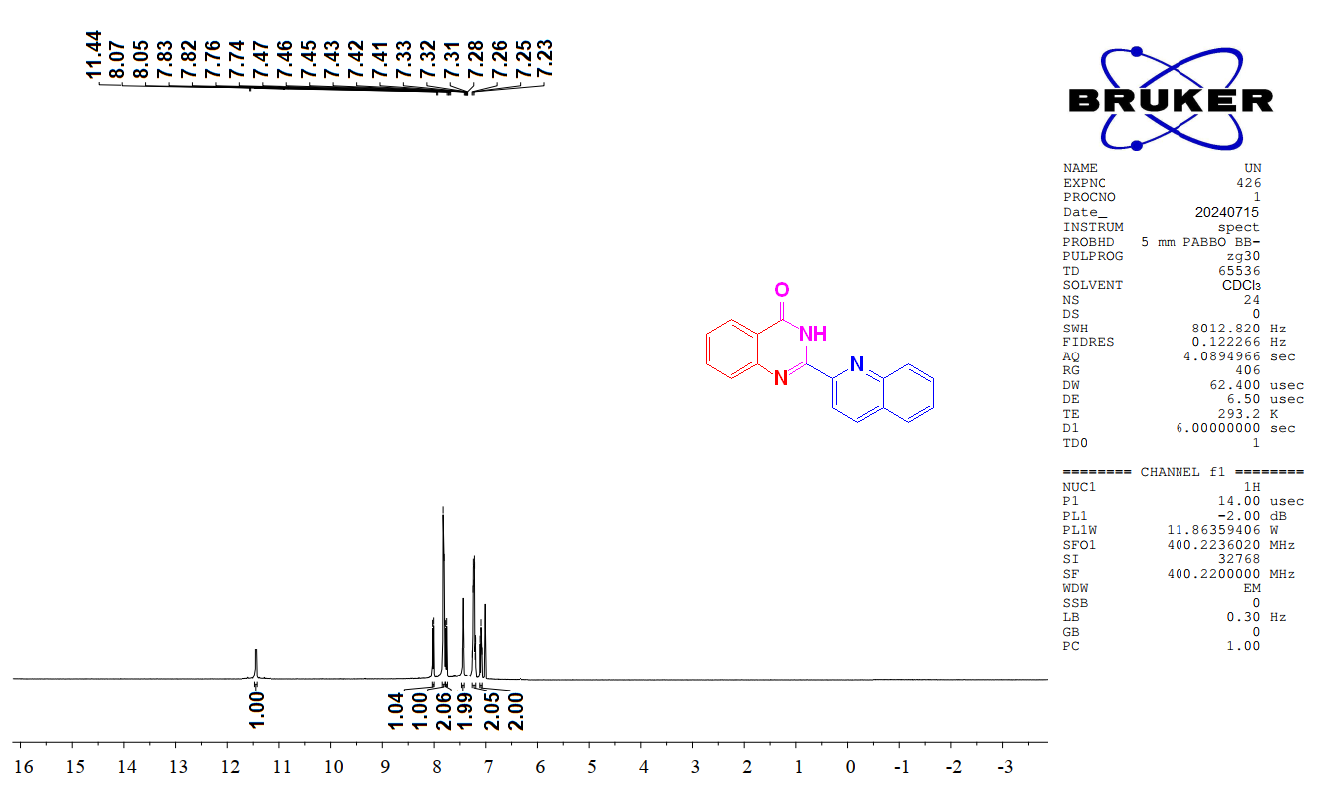


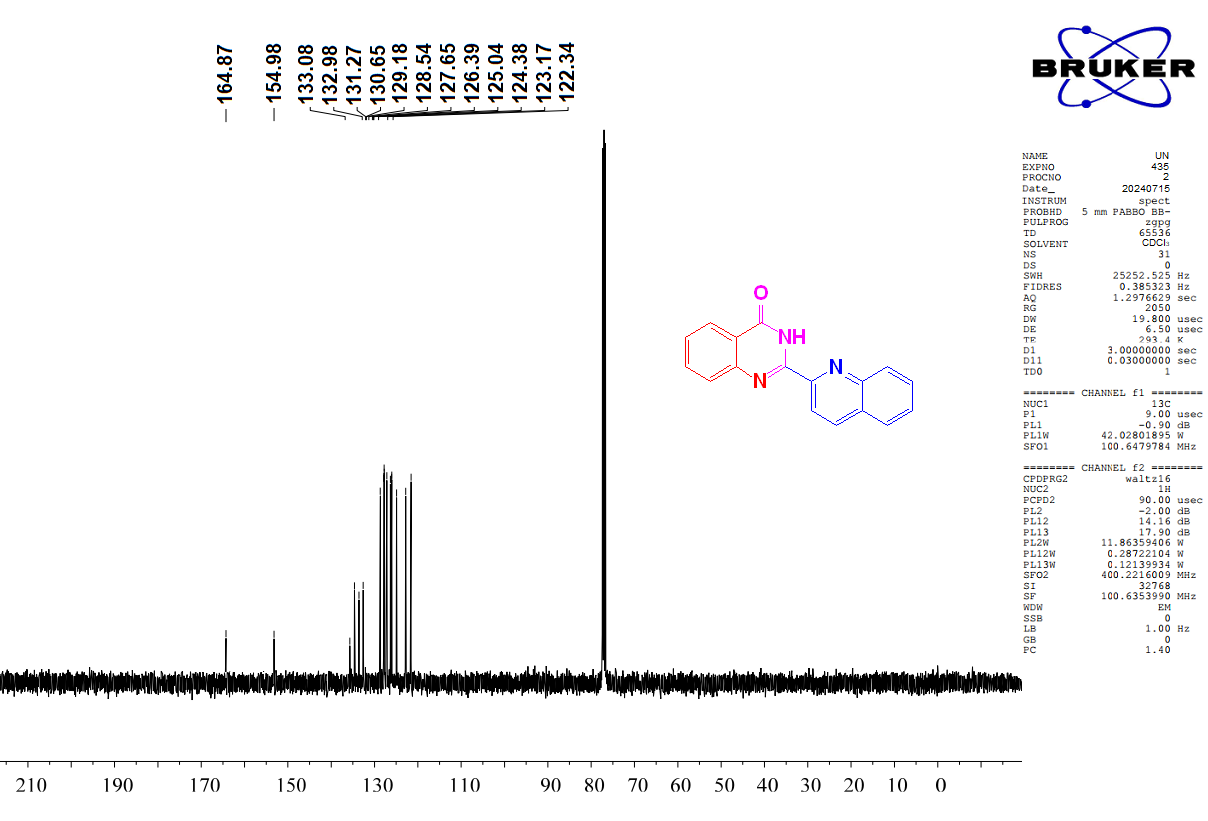


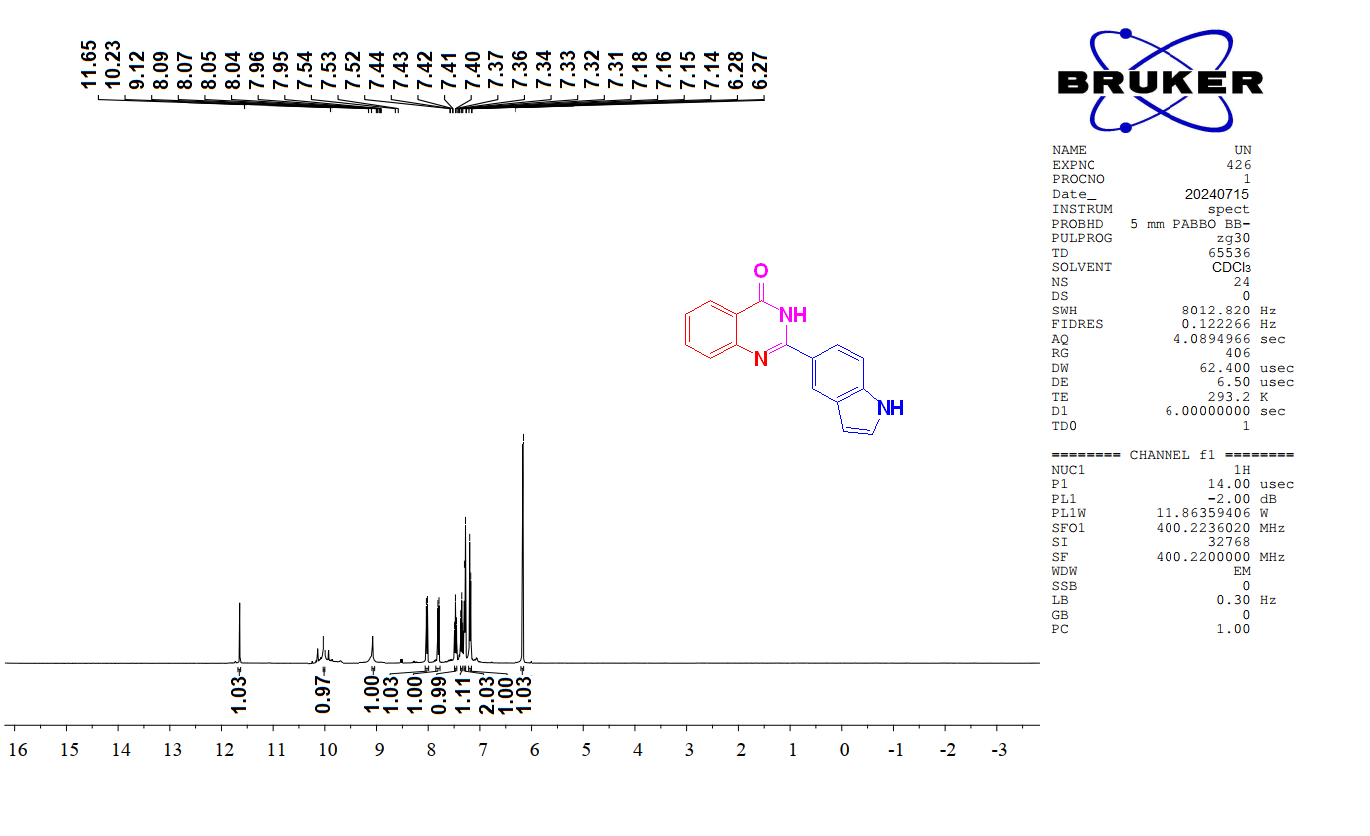


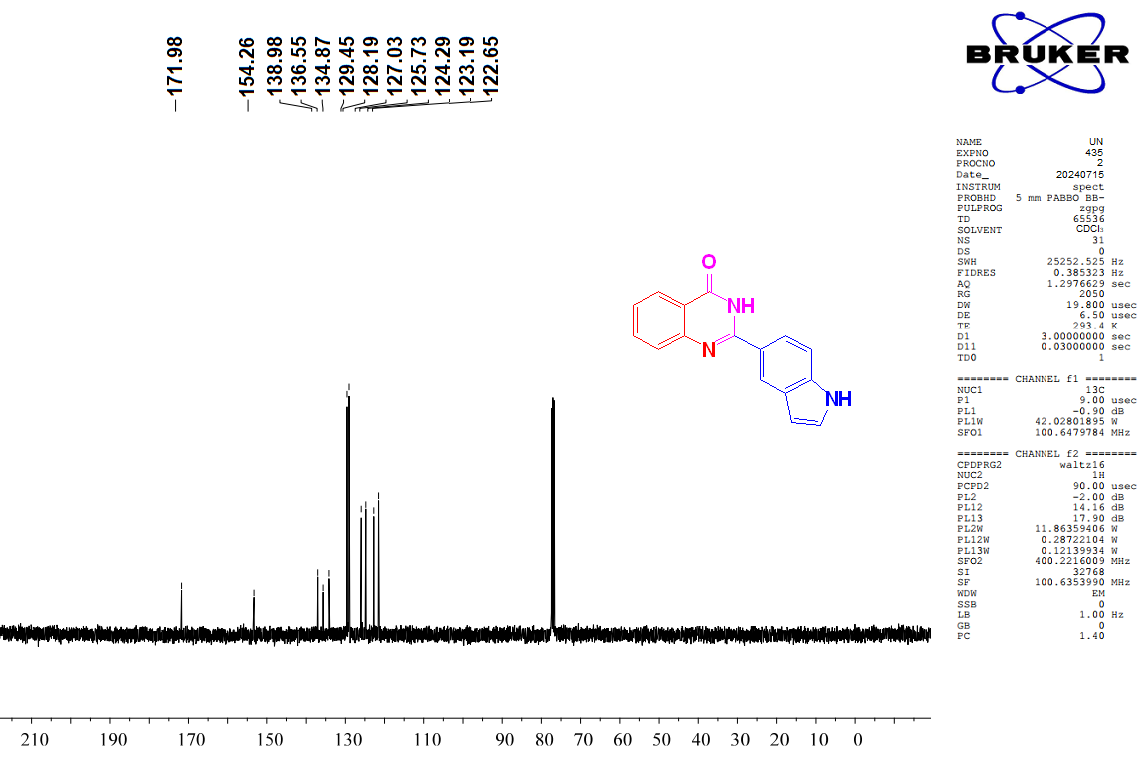

Supplement: Supplementary file 1 [file Table1.docx]
